# Novel Molecular Subtyping Scheme Based on In Silico Analysis of Cuproptosis Regulator Gene Patterns Optimizes Survival Prediction and Treatment of Hepatocellular Carcinoma

**DOI:** 10.3390/jcm12185767

**Published:** 2023-09-05

**Authors:** Heng Jiang, Hao Chen, Yao Wang, Yeben Qian

**Affiliations:** 1 Department of General Surgery, The First Affiliated Hospital of Anhui Medical University, Hefei 230022, China; 2 Department of Emergency Surgery, The First Affiliated Hospital of Anhui Medical University, Hefei 230022, China; 3 Department of Digestive Endoscopy, The First Affiliated Hospital with Nanjing Medical University, Nanjing 210029, China

**Keywords:** hepatocellular carcinoma, cuproptosis, genomic instability, senescence, the Cancer Genome Atlas program

## Abstract

Background: The liver plays an important role in maintaining copper homeostasis. Copper ion accumulation was elevated in HCC tissue samples. Copper homeostasis is implicated in cancer cell proliferation and angiogenesis. The potential of copper homeostasis as a new theranostic biomarker for molecular imaging and the targeted therapy of HCC has been demonstrated. Recent studies have reported a novel copper-dependent nonapoptotic form of cell death called cuproptosis, strikingly different from other known forms of cell death. The correlation between cuproptosis and hepatocellular carcinoma (HCC) is not fully understood. Materials and Methods: The transcriptomic data of patients with HCC were retrieved from the Cancer Genome Atlas-Liver Hepatocellular Carcinoma (TCGA-LIHC) and were used as a discovery cohort to construct the prognosis model. The gene expression data of patients with HCC retrieved from the International Cancer Genome Consortium (ICGC) and Gene Expression Omnibus (GEO) databases were used as the validation cohort. The Least Absolute Shrinkage and Selection Operator (LASSO) regression analysis was used to construct the prognosis model. A principal component analysis (PCA) was used to evaluate the overall characteristics of cuproptosis regulator genes and obtain the PC1 and PC2 scores. Unsupervised clustering was performed using the ConsensusClusterPlus R package to identify the molecular subtypes of HCC. Cox regression analysis was performed to identify cuproptosis regulator genes that could predict the prognosis of patients with HCC. The receiver operating characteristics curve and Kaplan–Meier survival analysis were used to understand the role of hub genes in predicting the diagnosis and prognosis of patients, as well as the prognosis risk model. A weighted gene co-expression network analysis (WGCNA) was used for screening the cuproptosis subtype-related hub genes. The functional enrichment analysis was performed using Metascape. The ‘glmnet’ R package was used to perform the LASSO regression analysis, and the randomForest algorithm was performed using the ‘randomForest’ R package. The ‘pRRophetic’ R package was used to estimate the anticancer drug sensitivity based on the data retrieved from the Genomics of Drug Sensitivity in Cancer database. The nomogram was constructed using the ‘rms’ R package. Pearson’s correlation analysis was used to analyze the correlations. Results: We constructed a six-gene signature prognosis model and a nomogram to predict the prognosis of patients with HCC. The Kaplan–Meier survival analysis revealed that patients with a high-risk score, which was predicted by the six-gene signature model, had poor prognoses (log-rank test *p* < 0.001; HR = 1.83). The patients with HCC were grouped into three distinct cuproptosis subtypes (Cu-clusters A, B, and C) based on the expression pattern of cuproptosis regulator genes. The patients in Cu-cluster B had poor prognosis (log-rank test *p* < 0.001), high genomic instability, and were not sensitive to conventional chemotherapeutic treatment compared to the patients in the other subtypes. Cancer cells in Cu-cluster B exhibited a higher degree of the senescence-associated secretory phenotype (SASP), a marker of cellular senescence. Three representative genes, *CDCA8*, *MCM6*, and *NCAPG2*, were identified in patients in Cu-cluster B using WGCNA and the “randomForest” algorithm. A nomogram was constructed to screen patients in the Cu-cluster B subtype based on three genes: *CDCA8*, *MCM6*, and *NCAPG2*. Conclusion: Publicly available databases and various bioinformatics tools were used to study the heterogeneity of cuproptosis in patients with HCC. Three HCC subtypes were identified, with differences in the survival outcomes, genomic instability, senescence environment, and response to anticancer drugs. Further, three cuproptosis-related genes were identified, which could be used to design personalized therapeutic strategies for HCC.

## 1. Introduction

Due to the high degree of malignancy, the prognosis of patients with advanced hepatocellular carcinoma (HCC) is usually poor. Due to the late diagnosis, most cases present beyond a cure, restricted to resection, transplantation, and radiofrequency ablation (RFA). The 5-year survival of patients with HCC is less than 30% [1]. Various studies have shown heterogeneity in the phenotypic and molecular features of HCCs [2]. Hence, the current focus is on understanding the heterogeneity of HCC, which will aid in the effective diagnosis, prognosis, and treatment of patients. Therefore, exploring the molecular subtypes of HCC at the transcriptomic level will assist in identifying clinically relevant gene signatures and prognosis factors [3,4]. Together, these results will aid in designing personalized and novel therapeutic strategies for the treatment of patients with HCC.

Recent studies have demonstrated a new atypical form of cell death called cuproptosis. According to a new study published by *Science*, the intracellular accumulation of copper (Cu) causes the aggregation of mitochondrial lipoylated proteins, thus destabilizing Fe-S cluster proteins, which results in a distinct form of cell death known as cuproptosis [5]. Elesclomol transports copper (Cu^2+^) from the outside to intracellular compartments of the cells by binding to copper (Cu^2+^). Cuproptosis primarily occurs due to the increased aggregation of copper and *FDX1*-mediated mitochondrial proteotoxic stress. *FDX1* facilitates the lipoylation (LA) and aggregation of enzymes, specifically dihy-drolipoamide S-acetyltransferase (DLAT), which controls the mitochondrial tricarboxylic acid cycle by reducing Cu^2+^ to Cu^+^. Simultaneously, *FDX1* also destabilizes the proteins in the Fe-S cluster. In addition to copper ionophores, copper importers such as SLC31A1 and exporters such as ATP7B control the sensitivity to cuproptosis by altering the intracellular Cu+ levels. Buthionine sulfoximine (BSO) promotes cuproptosis by depleting glutathione (GSH), which acts as a copper chelator and contains thiols, thereby preventing cuproptosis. Rotenone and antimycin A are electron transport chain complexes I/III, as well as mitochondrial pyruvate carrier (UK5099) inhibitors. They can inhibit the effects of elesclomol on cuproptosis [6].

Previous studies have demonstrated that copper is a transition metal essential for all living organisms and can act as a cofactor for several enzymes [7]. In vivo studies have shown that copper can be toxic if it exceeds a certain threshold [8]. The liver plays an important role in maintaining copper homeostasis. Further, many diseases like metabolic disorders and cancers are associated with copper homeostasis [9]. Several studies have shown a significant increase in copper levels in patients with cancer compared to healthy controls [10,11,12,13]. Copper is a cofactor for several enzymes and a key regulator for several signaling pathways associated with tumorigenesis, which influences cell proliferation and promotes angiogenesis and metastasis [14,15]. It has been established that copper plays a pivotal role in tumor progression; hence, several copper-based anticancer agents, including copper chelators and copper ionophores, are currently being investigated [16,17]. Dithiocarbamates are a subclass of metal-chelating compounds; they interact with metal ions to create metal complexes and are used in cancer treatment to target the undecaprenyl pyrophosphate synthase (UPP). Pyrrolidine dithiocarbamate is a member of the dithiocarbamate family, which binds to copper to inhibit cancer-specific proteasomes and trigger cell death in breast and prostate cancers [18].

Therefore, cuproptosis could be targeted for the treatment of patients with HCC. Hence, exploring the heterogeneity of cuproptosis in HCC may aid in the prognosis and development of therapeutic strategies for HCC. In Supplementary Figure S1, the workflow of our study is depicted.

## 2. Materials and Methods

### 2.1. Data Acquisition and Processing

Data on the clinical information, somatic mutations, and RNA expression of 423 tissue samples from 371 patients with HCC were obtained from The Cancer Genome Atlas (TCGA)-LIHC on the GDC website (https://portal.gdc.cancer.gov/ in 1 March 2022). TCGA-LIHC samples were used as the discovery cohort [19]. Further, clinical information and transcriptomic profiles of patients with HCC were retrieved from databases like the International Cancer Genome Consortium and Gene Expression Omnibus. The downloaded online Uniform Resource Locators (URLs) were “ICGC; https://dcc.icgc.org/ in 1 March 2022” and “GEO; http://www.ncbi.nlm.nih.gov/geo/ in 1 March 2022”, respectively. The information on the ICGC-LIRI-JP cohort was obtained from the ICGC database, and the datasets GSE36376, GSE102079, GSE76427, and GSE62061 were retrieved from the GEO database, which were used as the validation cohorts [20,21].

A previous study described ten genes associated with cuproptosis regulation, including *FDX1*, *LIAS*, *LIPT1*, *DLD*, *DLAT*, *PDHA1*, *PDHB*, *MTF1*, and *GLS* (also known as cuproptosis regulator genes), which were used for a subsequent analysis [5]. Further, senescence-associated secretory phenotype (SASP)-associated genes were used for further analysis, as described by Coppe et al. The genes significantly altered in the pre-senescent and senescent states are listed in Supplementary Table S1 [22].

### 2.2. Identification of Cuproptosis Subtypes

We employed the ‘ConsensusClusterPlus’ R package to investigate the expression patterns of cuproptosis regulator genes via unsupervised hierarchical clustering [23]. The cuproptosis subtypes were identified using the following parameters: 50 reps, 0.8 pItem, 1 pFeature, and the Euclidean distance. Unsupervised hierarchical clustering was performed for all factors closely associated with SASP. The parameters used were the same as those used to obtain different senescent subtypes in the TCGA-LIHC cohort.

### 2.3. Co-Expression Network Construction

We constructed co-expression networks by employing the R package “Weighted Gene Co-Expression Network Analysis” (WGCNA) [24,25]. We obtained the expression profiles of 2207 differentially expressed genes between HCC and normal tissues from the Gene Expression Profiling Interactive Analysis (GEPIA) database. The selection criteria included |Log2FC| > 1 and FDR < 0.01. These genes were then used as input files for the WGCNA analysis. To ensure the quality of the data used in our analysis, we calculated the median absolute deviation (MAD) for all genes. Subsequently, we removed 50% of the genes with low MAD values. We then employed the ‘goodSamplesGenes’ function in the WGCNA R package to eliminate any potential outlier samples and genes. Initially, Pearson’s correlation matrices for all pair-wise genes were established using the average linkage method. Then, a power function was applied to construct a weighted adjacency matrix. The power function used was defined by A_ab_ = |C_ab_|^β.

C_ab_ = Pearson’s correlation between gene a and gene b; A_ab_ = adjacency between Gene a and Gene b

A soft-thresholding parameter β was used to improve the strong gene correlations and penalize weak correlations. The adjacency matrix was transformed into the topological overlap matrix (TOM). The WGCNA R package’s “pickSoftThreshold” algorithm was used to select β = 10.

The power value of 10 was chosen for transforming the adjacency matrix into a topological overlap matrix (TOM). Subsequently, the genes were grouped into modules based on similar expression patterns using an average linkage hierarchical clustering algorithm. The grouping criteria included a minimum size of 30 genes per module and a sensitivity level of 3 for deep splitting. Further, the modules were merged if the distance between the modules was equal to or less than 0.25 (a minimum module merge cut height = 0.25), and a total of four modules were finally obtained.

To assess the correlations between gene modules and clinical features, we employed two methods. First, we calculated the module eigengene (ME) by deriving the first principal component of each gene module, which reflects the overall expression patterns of all genes within the module. The correlations between the MEs and clinical features were calculated to identify the gene modules associated with the cuproptosis subtypes. In addition, a linear regression analysis was conducted to examine the relationship between the gene expression and clinical features. The gene significance (GS) was determined by calculating the *p*-value (lgP) of each gene. To integrate the cuproptosis subtypes of interest into the co-expression network, the module significance (MS) was calculated as the average of the GS for all genes in each module.

### 2.4. Pathway and Process Enrichment Analyses

A web-based tool, Metascape, is utilized to estimate the similarities of membership between enriched items and classify them into different clusters. It was used to perform the pathway enrichment analysis [26]. The terms with *p* < 0.01, a minimum count of 3, and an enrichment factor >1.5 were considered significantly enriched. The accumulative hypergeometric distribution was used to calculate the *p*-value, and the q-value was calculated using the Benjamini–Hochberg procedure. To perform hierarchical clustering, we set the similarity metrics as kappa scores of 4 and identified clusters with a similarity >0.3. Representative enriched terms were displayed for each cluster based on the ones with the lowest *p*-values.

Twenty-two biological processes from previous studies and the Kyoto Encyclopedia of Genes and Genomes (KEGG) database were downloaded for a subsequent analysis and are listed in Supplementary Table S2 [27]. To estimate the enrichment scores of the biological processes for all samples, we utilized Single-sample Gene Set Enrichment Analysis (GSEA).

### 2.5. Drug Sensitivity Prediction

The ‘pRRophetic’ R package was used to predict drug responses and perform ridge regression to calculate the half-maximal inhibitory concentration (IC_50_) of drugs for each patient [28]. Data obtained from the Genomics of Drug Sensitivity in Cancer (GDSC) database were used for internal cross-validation, which was performed through 10-fold cross-validation [29]. Proteins translated by the hub genes were the subject of this investigation, and their crystal structures were retrieved from the Research Collaboratory for Structural Bioinformatics Protein Data Bank (www.rcsb.org/pdb/home/home.do in 1 March 2022). Additionally, we obtained the 3D structures of the small molecule drugs from the PubChem database, which can be accessed at https://www.ncbi.nlm.nih.gov/pccompound in 1 March 2022. To perform molecular docking, Autodock Vina was utilized. The process involved three steps: protein and ligand preparation, grid setup, and compound docking. The identification of the best pose included the consideration of both the docking score and the rationality of the molecular conformation.

### 2.6. Machine Learning for the Candidate Cuproptosis Subtype-Specific Gene Signature (CSGS)

The ‘glmnet’ and ‘randomForest’ R packages were used to perform the least absolute shrinkage and selection operator COX (LASSO-COX) regression analysis. The randomForest algorithm was used to screen for candidate CSGSs using the TCGA-LIHC cohort [30,31]. We utilized the LASSO-COX regression analysis to compress the insignificant coefficients to zero. The penalized function was used for reducing the dimensions of the feature space vector [32]. The final importance of the features and genes was determined using randomForest algorithms by taking into account the average importance of each feature in each iteration. The importance of features and genes greater than 5 were used for the downstream analysis [33]. For further analysis, we used the results from the intersection of the two methods.

### 2.7. Protein Expression of CSGSs Using the Human Protein Atlas (HPA) Database

The HPA database provides information on all proteins expressed by normal or tumor tissues obtained by integrating data from various omics technologies. The protein expression of CSGSs in normal and HCC tissues was retrieved from the HPA database [34].

### 2.8. Statistical Analyses

Statistical analyses were performed using R Studio software version 4.0.4 [35]. The nonparametric Wilcoxon test and Kruskal–Wallis test were used to calculate the *p*-values. The nonparametric Wilcoxon test was used for comparing the two groups, while the Kruskal–Wallis test was used for multiple comparisons. The chi-square test was used to examine the categorical variables. *p* < 0.05 was considered statistically significant. The Kaplan–Meier (KM) survival analysis and the log-rank test were used to calculate the overall survival (OS) of patients in different subgroups [36]. To predict the efficacy of the prognosis model, a receiver operating characteristic (ROC) curve was utilized [37]. Variables were analyzed for correlations using Pearson’s correlation analysis [38]. 

## 3. Results

### 3.1. Diagnostic and Prognostic Role of Cuproptosis Regulator Genes in Patients with HCC

**Figure 1:**
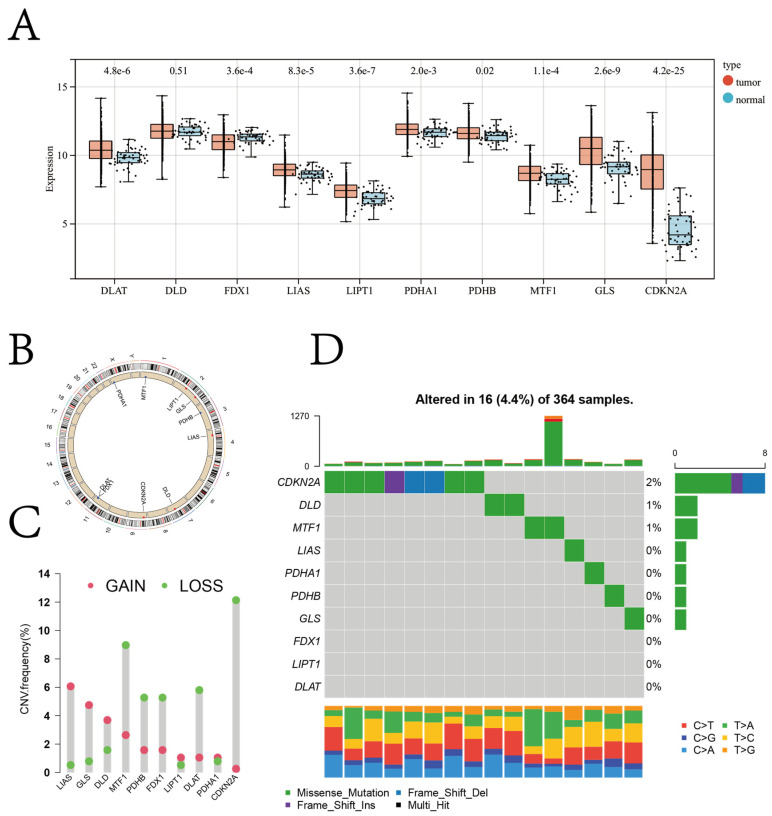
(**A**) Relative expression of cuproptosis regulator genes in the tissues from patients with HCC and healthy controls (red color represents tumor tissue, and green color represents normal tissue; the *p*-values are shown above the boxplots). (**B**) Chromosomal locations of cuproptosis regulator genes. (**C**) The frequency CNV of cuproptosis regulator genes in the TCGA-LIHC cohort. The column height represents the alteration frequency (red indicates copy number gain, whereas green indicates copy number loss). (**D**) Waterfall (oncoplot) plot of the cuproptosis regulator genes in the TCGA-LIHC cohort.

The gene expression data of 373 patients with HCC and 50 healthy controls retrieved from TCGA were analyzed using the Kruskal–Wallis test. The results revealed an increase in the expression of most cuproptosis regulator genes in patients with HCC compared to healthy controls, except for *FDX1* expression (Figure 1A).

The chromosomal location of the cuproptosis regulator genes and copy number variation (CNV) regions are shown in Figure 1B. In the TCGA-LIHC cohort, the CNV loss was significantly high in genes like *CDKN2A* and *MTF1*, whereas the CNV gain was significantly high in genes like *LIAS* and *GLS* (Figure 1C). A low mutation frequency was observed in the cuproptosis regulator genes in the TCGA-LIHC cohort (4.4%; Figure 1D).

**Figure 2:**
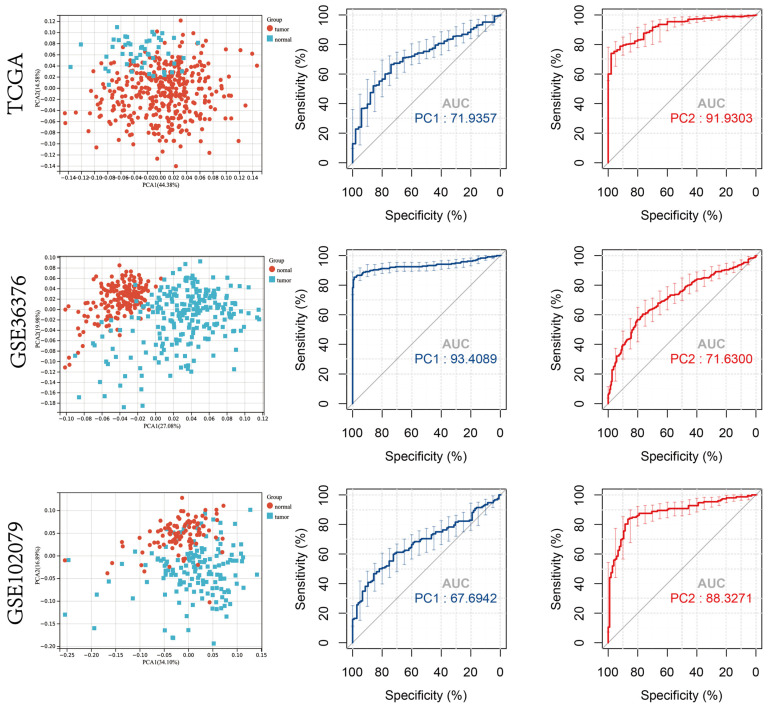
Principal component analysis (PCA) of expression patterns of cuproptosis regulator genes. ROC curve was used to determine the diagnostic accuracy of PC1 and PC2 in patients from the TCGA cohort and GSE36376 and GSE102079 datasets.

A principal component analysis (PCA) was performed on the expression profiles of the ten cuproptosis regulator genes [39]. The gene expression profiles were reduced to a two-dimensional space (PCA1 and PCA2) for visualization. The ROC curve was used to determine the diagnostic values of PC1 and PC2 in distinguishing patients with HCC and healthy controls. In the TCGA-LIHC cohort, the area under the ROC curve (AUC) value for PC1 was 0.72 and PC2 was 0.92. Further, the diagnostic ability of the ten cuproptosis regulator genes in distinguishing patients with HCC and healthy controls was validated on the GSE36376 and GSE102079 datasets using PCA, and the results obtained were consistent with the TCGA-LIHC cohort (Figure 2).

**Figure 3:**
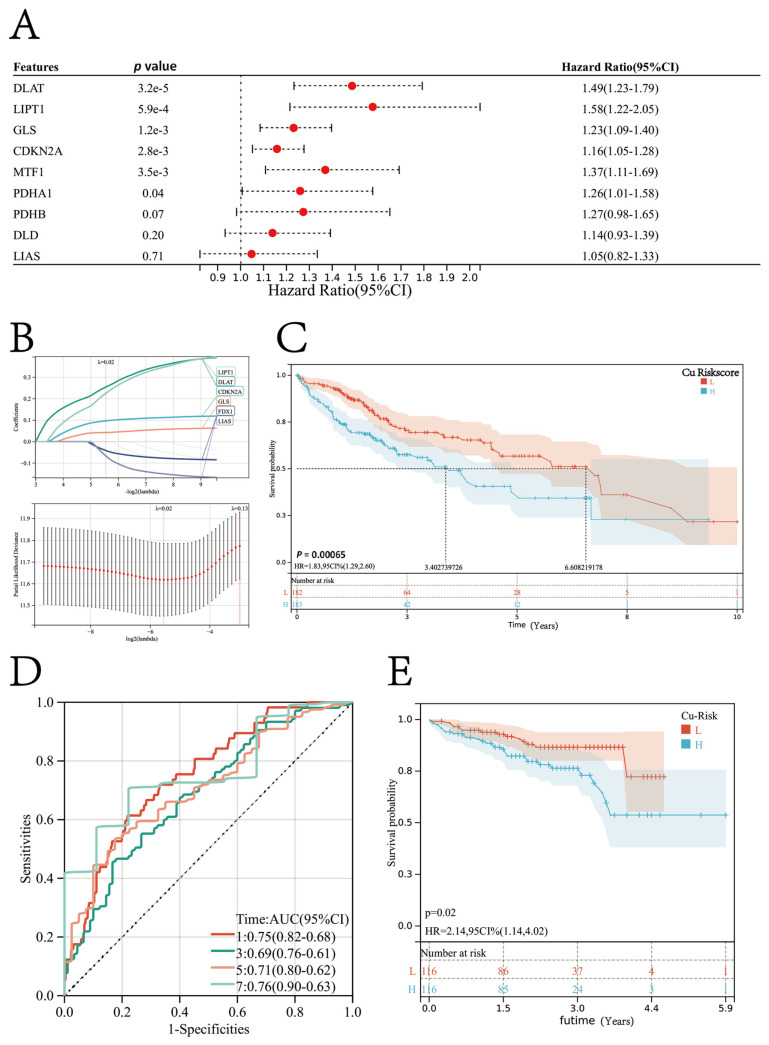
(**A**) Univariate Cox regression analysis used to determine the prognostic ability of cuproptosis regulator genes. (**B**) Riskscore prognosis model was constructed using a LASSO regression analysis. (**C**) The patients in the TCGA-LIHC cohort were classified into the high-risk or low-risk group based on the median risk score calculated by the prognosis model. The Kaplan–Meier survival curve shows that a high-risk score indicates a poor patient prognosis (HR= 1.83; log-rank test *p* = 0.00065). (**D**) The ROC curve of the prognosis model predicts the 1-, 3-, 5-, and 7-year OS of patients. (**E**) The Kaplan–Meier survival curves for patients with HCC in the independent validation cohort (ICGC-LIRI-JP cohort).

The overall survival (OS) data of the patients obtained from the TCGA database showed that the longest follow-up duration was 10.07 years, and the average follow-up duration was 2.39 years. A univariate regression analysis was performed to study the prognostic value of cuproptosis regulator genes in predicting the OS of the patients (Figure 3A). Of the ten cuproptosis regulator genes, six genes, including *FDX1*, *LIPT1*, *DLAT*, *PDHA1*, *MTF1*, *GLS*, and *CDKN2A*, were risk factors for a HCC prognosis. Based on the expression profiles of all ten cuproptosis regulator genes, six genes, including *FDX1*, *LIPT1*, *DLAT*, *PDHA1*, *MTF1*, *GLS*, and *CDKN2A*, were identified using the LASSO regression algorithm. These six genes were used to construct the prognosis model (Figure 3B). The formula for the prognosis model was:

Cu-Riskscore = (0.0971 × *CDKN2A* exp.) + (0.2549 × *DLAT* exp.) + (−0.0414 × *FDX1* exp.) + (0.0428 × *GLS* exp.) + (−0.0667 × *LIAS* exp.) + (0.2257 × *LIPT1* exp).

The risk score of each patient in the TCGA-LIHC cohort was calculated and ranked using the formula. The patients were then classified into the high-risk or low-risk Cu-Riskscore group based on the median value. According to the K–M survival analysis, patients with a high Cu-Riskscore had a poor prognosis. Therefore, a high Cu-Riskscore indicates the poor prognosis of patients with HCC (Log-rank *p* = 0.00065; Figure 3C). The OS of patients in the high Cu-Riskscore group was significantly less compared to patients in the low Cu-Riskscore group (median OS: 3.40 versus 6.60 years). The ROC curve for 1-year OS indicated that the prognosis model constructed using six genes had an outstanding performance in predicting the OS, with an AUC value of 0.75 (Figure 3D).

The prognosis model was further validated on the ICGC-LIRI-JP cohort. The data retrieved from the ICGC revealed that the longest follow-up was conducted for 5.92 years, and the average follow-up duration was 2.18 years. In the ICGC-LIRI-JP cohort, the prognosis of patients with a high Cu-Riskscore was poor (HR = 2.14, 95% CI = 1.14–4.02; Figure 3E). Hence, the Cu-Riskscore could be used to predict the prognosis of patients.

To facilitate user-friendly graphical interfaces, a nomogram was constructed with the Cu-Riskscore and tumor stage (Supplementary Figure S2A). The calibration curves revealed the accuracy of the nomogram in predicting the 1-, 3-, and 5-year OS of patients with HCC in the TCGA-LIHC (Supplementary Figure S2B).

### 3.2. Cuproptosis Subtypes in HCC

**Figure 4:**
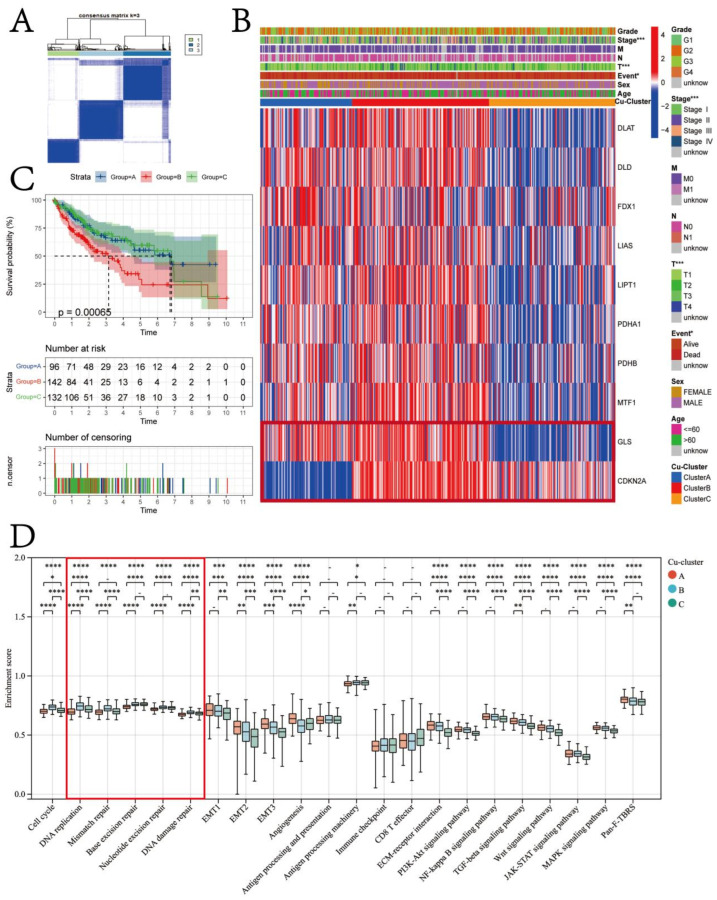
Identification of different cuproptosis subtypes in the TCGA-LIHC cohort. (**A**) Consensus matrices of the TCGA-LIHC cohort for k = 3. (**B**) The heatmap shows the expression of cuproptosis regulator genes among the three cuproptosis clusters. (**C**) Kaplan–Meier survival analyses show the OS of patients from the TCGA-LIHC cohort in different cuproptosis clusters. (**D**) Enrichment scores of 22 known biological processes related to tumor progression in different cuproptosis clusters. The asterisks represent the statistical *p*-values (* *p* < 0.05; ** *p* < 0.01; *** *p* < 0.001; **** *p* < 0.0001).

Using the ‘ConsensusClusterPlus’ R package, we conducted the unsupervised clustering of patients with HCC based on the expression of ten cuproptosis regulator genes. As a result, we classified them into three different subtypes, namely Cu-cluster A, Cu-cluster B, and Cu-cluster C (Figure 4A). Significant differences in the expression of the ten cuproptosis regulator genes were observed in the patients in the three clusters (Figure 4B).

Furthermore, the K–M survival analysis was performed to analyze the OS of patients with HCC. The K–M survival analysis revealed that the median OS of patients in Cu-cluster B was 3.05 years, which was significantly lower compared to the median OS of patients in Cu-cluster A and Cu-cluster C (log-rank test *p* = 0.00065; Figure 4C). The heatmap of the three cuproptosis clusters based on the expression pattern of ten cuproptosis regulator genes revealed distinct expression patterns of genes in the patients in the three clusters (Figure 4B). The expression of most cuproptosis regulator genes was low in patients in Cu-cluster C (Supplementary Figure S3A). A significant difference in the tumor stage and T stage among patients in the three clusters was observed based on the chi-square test results (Supplementary Figure S3B,C). The patients in Cu-cluster B exhibited high expression levels of GLS and CDKN2A, which was associated with a poor prognosis. Notably, GLS and CDKN2A were expressed at high and low levels, respectively, in Cu-clusters A and C. Therefore, we next assessed if the difference in *GLS* and *CDKN2A* expression could contribute to the difference in patient prognosis. The patients were divided based on the median *GLS* and *CDKN2A* expression levels into *GLS* (low) *CDKN2A* (low), *GLS* (low) *CDKN2A* (high), *GLS* (high) *CDKN2A* (low), and *GLS* (high) *CDKN2A* (high) groups. The results revealed that patients with high *GLS* and *CDKN2A* expression had poor prognoses (Supplementary Figure S3D,E).

The validation cohort (ICGC-LIRI-JP and GSE76427) underwent an unsupervised clustering analysis using the same workflow. This resulted in the classification of patients in the validation cohort into three clusters, as shown in Supplementary Figure S3F,G. The data obtained from the GSE76427 dataset revealed that follow-up was conducted for 7.76 years, and the average follow-up duration was 1.82 years, a significant difference in the prognosis of patients in the three clusters (Supplementary Figure S3H,I). 

In order to comprehend the basis of the variations in patient prognosis within the TCGA-LIHC cohort, we conducted a comparison of the enrichment scores for 22 biological processes across the three cuproptosis clusters among the patients (as illustrated in Figure 4D). The biological processes associated with the DNA damage repair function had the highest median enrichment scores in the patients in Cu-cluster B (Figure 4D). Moreover, a significant increase in the enrichment scores of processes like epithelial–mesenchymal transition (EMT) and angiogenesis was observed in patients in Cu-cluster A. The enrichment score for the ECM–receptor interaction was lowest for the patients in Cu-cluster C.

**Figure 5:**
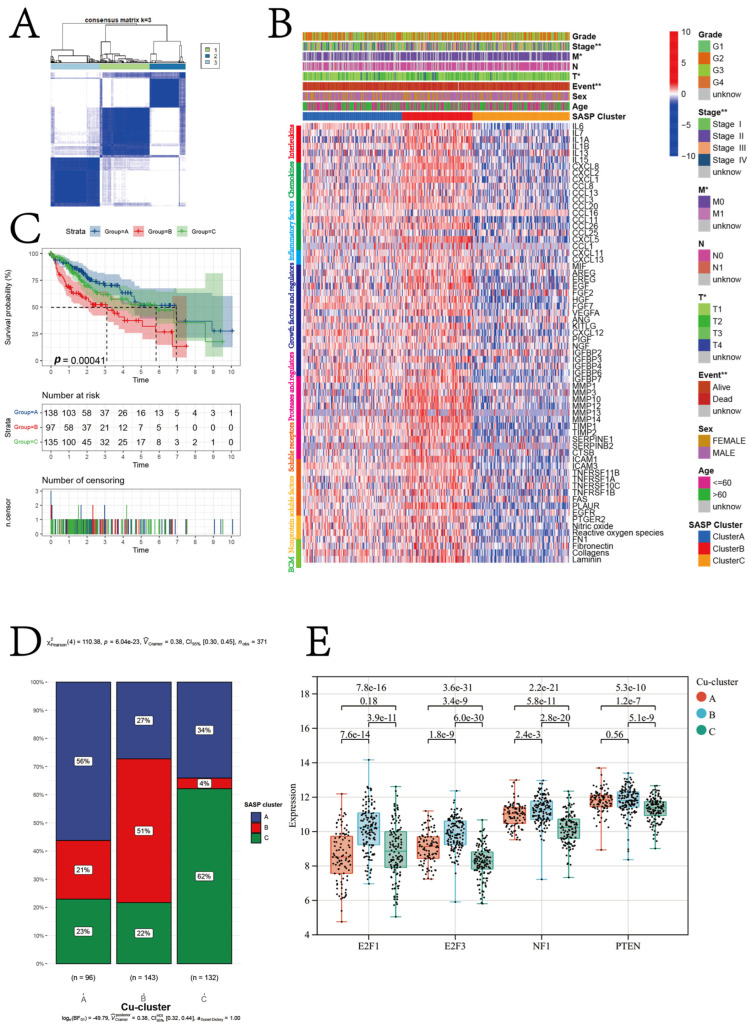
Identification of different SASP subtypes in the TCGA-LIHC cohort. (**A**) Consensus matrices of the TCGA-LIHC cohort for k = 3. (**B**) The heatmap shows the expression levels of SASP regulator genes among the three SASP subtypes. (**C**) K–M survival analysis shows the OS of patients from the TCGA-LIHC cohort in different SASP subtypes. (**D**) The proportion of different SASP subtypes in the three cuproptosis clusters and *p*-values was calculated by the chi-square test. (**E**) Boxplot for the relative expression of four senescence-associated genes in patients in the three cuproptosis subtypes, and the *p*-values are above the boxplots.

We performed unsupervised hierarchical clustering on 66 SASP regulator genes. The results revealed that the patients in the TCGA-LIHC cohort were classified into three senescent subtypes (SASP-clusters): intermediate, suppressive, and stimulatory (Figure 5A,B). Figure 5B illustrates the pattern of expression for SASP regulator genes in patients belonging to the TCGA-LIHC cohort. A significant difference in the prognosis of patients was observed between the three SASP-clusters (*p* = 0.0004; Figure 5C). A significantly higher proportion (56%) of the intermediate SASP subtype was observed in the patients belonging to Cu-cluster A, as determined by the chi-square test (*p* < 0.0001). Moreover, the patients belonging to Cu-cluster B showed a significantly higher proportion of the stimulatory SASP subtype (determined by the chi-square test; *p* < 0.0001). In patients belonging to Cu-cluster C, a high proportion of the suppressive subtype was observed, along with a significantly low proportion of the stimulatory SASP subtype (as illustrated in Figure 5D and determined by the chi-square test; *p* < 0.0001). The expression of four senescence-associated genes was compared among the patients in the three Cu clusters for further validation. The results revealed a significant increase in the expression of four senescence-associated genes in the patients in Cu-cluster B (Figure 5E).

A significant difference in enrichment scores of the biological processes associated with DNA damage repair function was observed among patients in the three cuproptosis clusters. Hence, the mutation frequency of the top 15 genes in different cuproptosis clusters was analyzed. The waterfall plot revealed the prominent activation of TP53 mutation in patients in Cu-cluster B (Supplementary Figure S4A). Further, the homologous recombination deficiency (HRD) and loss of heterozygosity (LOH) scores of all patients in the TCGA-LIHC cohort were obtained from the study by Vésteinn et al. (Supplementary Table S3). The patients in Cu-cluster B had significantly high HRD and LOH scores. However, no significant differences in the HRD and LOH scores were observed in the patients in Cu-clusters A and C (Supplementary Figure S4B,C). In addition, we compared the expression patterns of 16 HRD-related genes across the patients in all Cu-clusters. Consistent with our hypothesis, the patients in Cu-cluster B showed higher expression levels of most HRD-related genes, as shown in Supplementary Figure S4B. Furthermore, the expression levels of the HRD-related genes were observed to be higher in patients with HCC in comparison to healthy controls, with the exception of FANCC.

### 3.3. Therapeutic Potential of Cuproptosis Subtypes in HCC

Anticancer drugs, including Sorafenib, Lapatinib, Erlotinib, Axitinib, and Gefitinib, have been approved by the Food and Drug Administration to treat patients with HCC. Rucaparib (AG.014699) is a PARP inhibitor, and a correlation was observed between HRD and a sensitivity to PARP inhibitors. Rebemadlin (Nutlin.3a) can inhibit the MDM2–p53 interaction, thereby inducing p53-mediated apoptosis. Serdemetan (JNJ.26854165) is an E3 ligase *HDM2* inhibitor and can promote the apoptosis of p53 wild-type cells.

**Figure 6:**
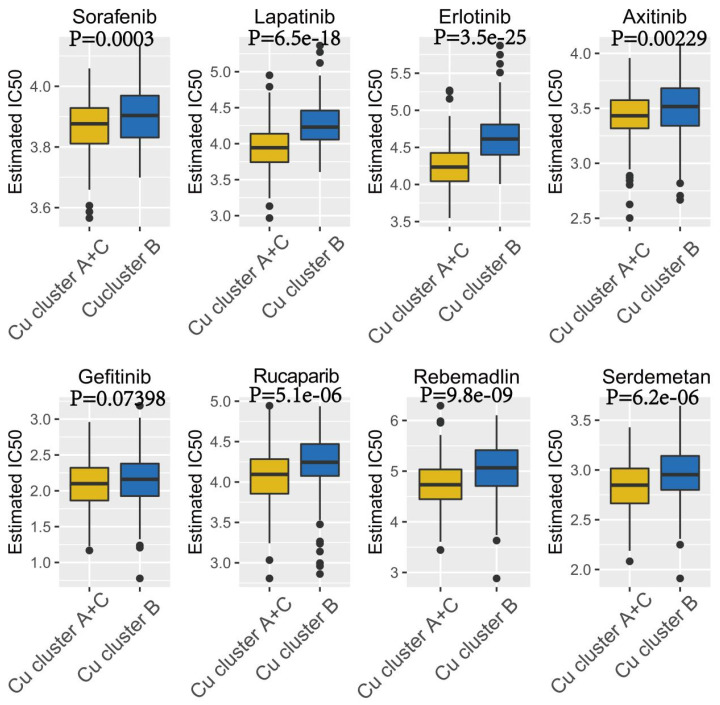
The “pRRophetic” R package was used to calculate the IC50 values of anticancer drugs like Sorafenib, Lapatinib, Erlotinib, Axitinib, Gefitinib, Rucaparib (AG.014699), Rebemadlin (Nutlin.3a), and Serdemetan (JNJ.26854165) in patients in Cu-cluster B and Cu-clusters A and C.

Therefore, these eight anticancer drugs were selected, as the patients in the different cuproptosis clusters could respond differently to these drugs. The IC_50_ value was calculated using the ‘pRRophetic’ R package for the patients in the TCGA-LIHC cohort. The results revealed that the patients in Cu-cluster B were less sensitive to all eight anticancer drugs compared to the patients in Cu-clusters A and C (*p* < 0.05; Figure 6).

### 3.4. Identification of Candidate CSGSs using WGCNA and Machine Learning Techniques

As shown previously, the patients in Cu-cluster B were less sensitive to chemotherapy. The prognosis of the patients in Cu-cluster B was poor and had genomic instability. Hence, the WGCNA and machine learning techniques were used to explore the candidate CSGS.

**Figure 7:**
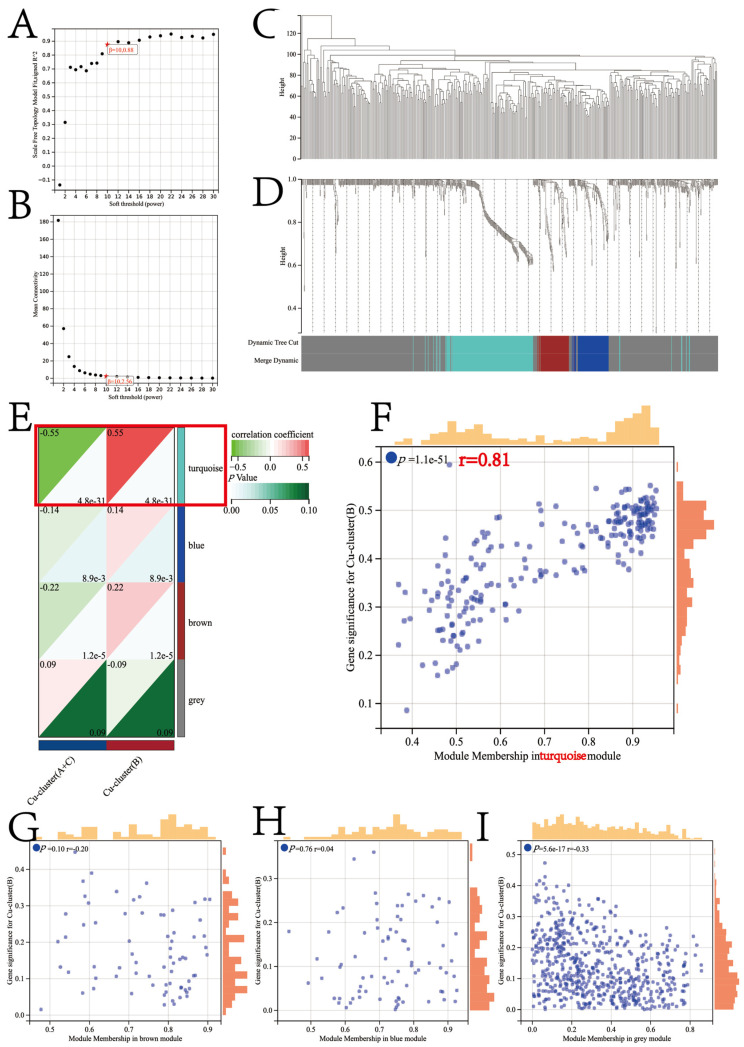
Determination of the soft threshold power in the WGCNA. (**A**) Analysis of the scale-free index for various soft threshold powers (β). (**B**) Analysis of the mean connectivity for various soft threshold powers. (**C**) Clustering dendrogram of 371 patients. Identification of modules closely associated with the cuproptosis subtypes. (**D**) Dendrogram of all differentially expressed genes clustered based on the measurement of dissimilarity (1-TOM). The color band shows the results obtained from the automatic single-block analysis. (**E**) Heatmap of the correlation between the module eigengenes and cuproptosis cluster of patients with HCC. A scatterplot of the gene significance (GS) for Cu-cluster B versus module membership (MM) in the turquoise module (**F**), brown module (**G**), blue module (**H**), and grey module (**I**).

Using the “WGCNA” R package and analyzing the expression patterns of 2207 genes in 371 HCC patients, we established the co-expression module. The independence and average connectivity degree were significantly affected by the power value, which was set to soft power 10 to construct a weighted adjacency matrix (Figure 7A,B). A total of 975 genes, including those in the grey module in the TCGA-LIHC cohort, were clustered into one of four co-expression modules, as demonstrated in the cluster analysis of the HCC patient samples (Figure 7C). The co-expression modules were visually distinguished by different colors in Figure 7D, and the genes clustered in each module are provided in Supplementary Table S4.

The correlation between the Cu-cluster B and turquoise modules was found to be significant (correlation coefficient = 0.55, *p* < 0.0001) according to the module–trait correlation heatmap shown in Figure 7E. The scatterplot of GS versus module membership (MM) for turquoise is illustrated in Figure 7F. The turquoise module exhibited a high correlation coefficient and smaller *p*-value in the correlation analysis (Figure 7G–I). Therefore, the turquoise module was identified as the characteristic module for Cu-cluster B, which consisted of 217 unique genes. A total of 102 genes in the turquoise module, meeting the criteria of |MM| > 0.8 and |GS| > 0.1, were screened as hub genes and are listed in Supplementary Table S5. Metascape was employed to perform a pathway enrichment analysis on the 102 hub genes, and the top 20 clusters with enriched representative terms (one term/cluster) are presented in detail in Supplementary Table S6 and Supplementary Figure S5A,B.

**Figure 8:**
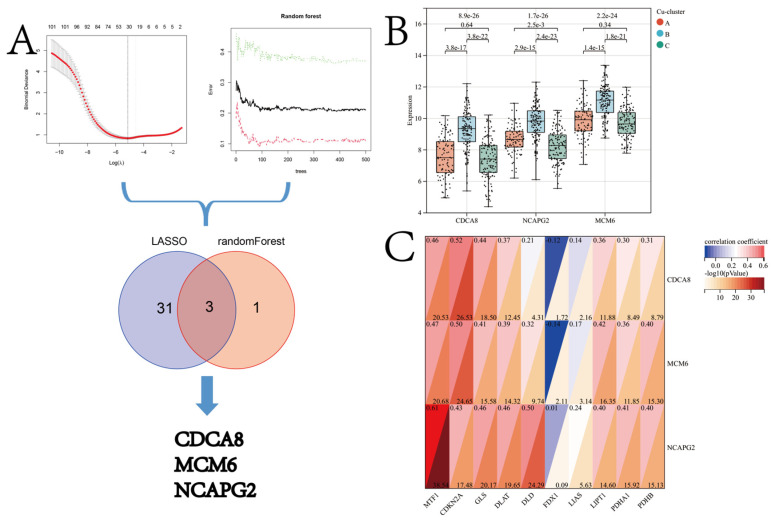
(**A**) Flow chart of the screening procedure. (**B**) Differences in expression of the CSGSs (CDCA8, MCM6, and NCAPG2) in patients in different cuproptosis clusters (*p*-values were calculated using the Kruskal–Wallis test). (**C**) Pearson’s correlation analysis was used to evaluate the correlation between CSGSs and cuproptosis regulator genes.

To screen for potential biomarkers between the patients in Cu-cluster B and Cu-clusters A and C of the TCGA-LIHC cohort, we employed two algorithms. After performing a LASSO regression analysis on the 102 genes from the turquoise module, we obtained a reduced set of 34 genes. These 34 genes were identified as diagnostic biomarkers, as shown in Figure 8A. The randomForest algorithm was used to identify four genes from 102 hub genes in the turquoise module (Figure 8A). Finally, the three overlapping genes, *CDCA8*, *MCM6*, and *NCAPG2*, identified using the two algorithms, were considered candidate CSGSs. The GEPIA database was used to study the expression of the CSGSs. The results revealed an increase in the expression of the CSGSs in tissues of patients with HCC compared to normal tissues. Further, the prognosis of patients with high CSGS expression was poor. Moreover, an increase in the expression of CSGS was observed in patients with advanced stage HCC. The expression patterns of the CDCA8, MCM6, and NCAPG2 proteins were retrieved from the HPA database. The immunohistochemistry results revealed that the expression of CDCA8, MCM6, and NCAPG2 was low in normal liver tissues but high in HCC tissues (Supplementary Figure S6A–C).

**Figure 9:**
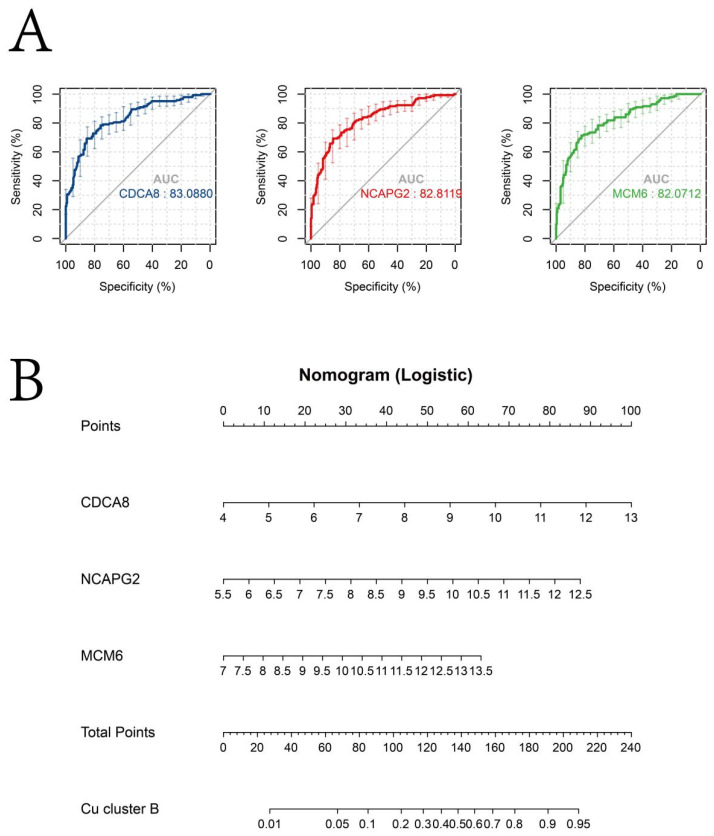
(**A**) The ROC curve shows the accuracy of CDCA8, MCM6, and NCAPG2 in predicting the diagnosis of patients in Cu-cluster B from the TCGA-LIHC cohort. (**B**) Construction of a nomogram based on the expression of CDCA8, MCM6, and NCAPG2 to identify patients in Cu-cluster B.

The Kruskal–Wallis test results revealed that the expression of *CDCA8*, *MCM6*, and *NCAPG2* was highest in the patients in Cu-cluster B (Figure 8B). Pearson’s correlation analysis revealed a moderate correlation between the CSGSs and cuproptosis regulators (Figure 8C). The diagnostic performance of CDCA8, MCM6, and NCAPG2 for discriminating patients in Cu-cluster B from the TCGA-LIHC cohort is demonstrated via a ROC analysis, yielding satisfactory results (Figure 9A). Moreover, a nomogram based on the aforementioned biomarkers is developed as a clinical tool to facilitate the identification of patients in Cu-cluster B (Figure 9B).

Furthermore, CSGS expression could predict the patient’s sensitivity to anticancer drugs like Lapatinib, Erlotinib, and Axitinib (Supplementary Figure S7A–C). Based on the median expression values of *CDCA8*, *MCM6*, and *NCAPG2*, the patients in the TCGA-LIHC cohort were divided into two groups. The IC_50_ of Lapatinib, Erlotinib, and Axitinib was low in patients with low *CDCA8*, *MCM6*, and *NCAPG2* expression. This indicates that *CDCA8*, *MCM6*, and *NCAPG2* could be used as novel indicators of a patient’s response to drugs like Lapatinib, Erlotinib, and Axitinib. These results were further validated by analyzing the Erlotinib resistance in patients from the GSE62061 dataset. The results revealed that *NCAPG2* expression could predict Erlotinib sensitivity in patients with HCC (Supplementary Figure S8A,B). Molecular docking was performed using Autodock Vina to check if Erlotinib could directly inhibit NCAPG2 expression. The results revealed that Erlotinib had a good binding affinity for NCAPG2, with a docking score of −7.2 kcal/mol (Supplementary Figure S8C,D). Erlotinib is a small-molecule *EGFR*-specific tyrosine kinase inhibitor. The 1-cCick Docking database (https://mcule.com/apps/1-click-docking/in 1 March 2022) revealed a docking score of –7.4 kcal/mol observed between Erlotinib and EGFR.

## 4. Discussion

Cuproptosis is a novel mechanism of regulated cell death; however, the roles of cuproptosis in HCC have not been well characterized [5]. In this study, we used various publicly available databases and bioinformatic tools to identify, as well as characterize, multiple cuproptosis regulator genes. We explored the distinct alteration/mutation patterns in cuproptosis regulator genes in patients with HCC. The proposed approach could facilitate the comprehension of the etiology and advancement of HCC, as well as contribute to the development of pioneering therapeutic and prognostic tactics for patients with HCC. 

A significant difference in the expression patterns of cuproptosis regulator genes was observed between tissues of healthy controls and patients with HCC; thus, the overall characterization of cuproptosis regulator genes could serve as a potential diagnostic biomarker for patients with HCC (Figure 2). In tissues of patients with HCC, the expression of most cuproptosis regulator genes was found to be upregulated. In contrast, the expression of FDX1 was found to be upregulated in healthy controls. Previous studies have shown that *FDX1* is a critical initiator of cuproptosis; however, the expression of *FDX1* is downregulated in various solid tumors [40,41]. Further, the CNV in different cuproptosis regulator genes was observed, which could be the primary cause for perturbations in the expression patterns of some cuproptosis regulator genes such as *FDX1*, *LIAS*, and *GLS*. Therefore, alterations in the CNV of cuproptosis regulator genes could be a potential underlying cause of cuproptosis heterogeneity in HCC [42]. 

We performed a further analysis to investigate if cuproptosis regulator genes could be used as prognostic biomarkers for HCC. Previous studies mostly concentrated on examining single regulator genes, neglecting the comprehensive characterization of the collective involvement of multiple cuproptosis regulator genes in HCC. Goh et al. [43] demonstrated the role of *DLAT* in cell proliferation, carbohydrate metabolism, and reprogramming in gastric cancers. A study by Sievers et al. [44] showed that the homozygous deletion of *CDKN2* could be used as a prognostic biomarker for meningiomas and an independent molecular biomarker for grading tumors. Ji et al. [45] demonstrated that *MTF1* is an oncogene and plays a role in the metastasis of ovarian cancers by promoting EMT. In this study, we performed a comprehensive analysis of the integrated roles of multiple cuproptosis regulator genes, unlike previous studies. Our results indicated that most cuproptosis regulator genes were the risk factors for the OS of patients with HCC, consistent with previous studies [46]. The LASSO regression analysis was used to create a novel prognosis model based on the Cu-Riskscore, and a nomogram was constructed based on both the Cu-Riskscore and tumor stage. The prognosis model constructed using the LASSO regression analysis was robust, and this is a widely recognized approach. In addition, fewer genes (*n* = 6) were included in our final prognosis model compared to previous studies (Supplementary Table S7). In addition, the prognostic model exhibited good performance in predicting the 1-year overall survival, as evidenced by an area under the curve (AUC) of 0.75. The prognosis model was constructed using only a few genes, which makes it easier to use in clinical settings. 

Following the analysis of cuproptosis regulator gene expression in patients with HCC, we classified patients into three distinct molecular clusters (Cu-cluster A, Cu-cluster B, and Cu-cluster C) based on the integrated role and expression pattern of various cuproptosis regulators. In addition, patients belonging to Cu-cluster B exhibited the most malignant form of HCC and had a poor prognosis. Interestingly, three Cu-clusters enriched 22 signaling pathways associated with tumorigenesis and cancer progression. Further, the genes in Cu-cluster B significantly enriched pathways related to DNA damage repair. This indicates higher genomic instability in patients in Cu-cluster B. Various studies have shown that genomic instability plays an important role in the development and progression of cancers. Further, the prognosis of patients with high genomic instability is also poor [47]. Thus, genomic instability could be an underlying factor associated with the poor prognosis of patients in Cu-cluster B. To further validate our results, the mutation frequency of the 15 most frequently mutated genes in HCC was analyzed in the patients in the three Cu-clusters. Mutations in *TP53* were detected in over 50% of patients in all the Cu-clusters. A significantly higher mutation in *TP53* was observed in the patients in Cu-cluster B compared to the patients in Cu-clusters A and C. TP53 is a cell cycle checkpoint gene that maintains genomic stability by inducing cell cycle arrest or the apoptosis of abnormal cells or cells harboring DNA damage that occurs during chromosome segregation [48,49,50]. Furthermore, the HRD and LOH scores were high in patients in Cu-cluster B. Hence, the HRD and LOH scores could be used as the scoring system to quantify the indices of genomic stability [51]. Additionally, the expression of all the HRD-related genes was high in the patients in Cu-cluster B. Together, these results further validated that the high genomic instability was one of the important features of the patients in Cu-cluster B. A recent in vitro study suggested that the levels of oxidative stress and DNA damage were induced and the DNA damage repair mechanism was impaired in the human neuroblastoma SH-SY5Y cell line after being incubated in a copper-containing solution (350 μM CuSO_4_) for 24 h [52]. The research of Hannah et al. revealed that the absence of trace elements, including copper, in the diet for 9 weeks led to a significant increase in inflammatory mediators in both the serum and liver, along with hepatic genomic instability [53]. These findings suggest that copper homeostasis plays a critical role in DNA damage and genome stability, highlighting the need for the further exploration of this mechanism. 

Aging causes decay of the nuclear genome [54]. Previous studies have demonstrated a high mutation burden in older individuals, which decreases tissue function and increases susceptibility to various age-related diseases [55]. Therefore, additional studies are required to explore if the genomic instability in the patients in Cu-cluster B was caused due to the presence of a high level of the senescence microenvironment. Senescent cells express and secrete a complex mixture of extracellular proteins and soluble factors, which is called SASP [56]. We evaluated the expression patterns of senescence-associated genes to study the senescence microenvironment. As expected, a high expression of senescence-associated genes was observed in the patients in Cu-cluster B. Previous studies have indicated that the accumulation of copper in human fibroblasts promotes the development of senescence. In cases of copper-induced premature senescence, ROS-induced p38^MAPK^ activation leads to an imbalance between DNA damage and repair. p38^MAPK^ is a potent inducer of replicative senescence in human proliferating cell types and is partly responsible for encoding genes related to antioxidant defense, such as Hsp70 and HO-1 [57]. Resveratrol, an anti-HCC drug, can attenuate CuSO_4_-induced cellular senescence by upregulating autophagy, maintaining protein homeostasis, and enhancing the cellular stress resistance [58]. Therefore, investigating the correlation between copper homeostasis and the senescence microenvironment in HCC can help identify potential therapeutic targets. *CDKN2A* is a cuproptosis regulator gene and can establish replicative senescence by activating the CDKN2A(p16)/RB signaling axis [59]. A previous study showed an increase in *DLAT* expression in B cells in older patients with chronic lymphocytic leukemia [60]. Further, a study by Yingying et al. suggested that an increased expression of GLS could enhance the production of the nicotinamide adenine dinucleotide phosphate (NADPH) and glutathione, thereby reducing oxidative stress. Notably, oxidative stress is an important cause of genomic instability in cells, which is characteristic of most cancer cells [61]. Together, these results indicated that both individual cuproptosis regulator genes and comprehensive characteristics of the cuproptosis subtypes were closely associated with senescence and genomic stability. Moreover, our prognosis model constructed using cuproptosis was found to be superior to most genome instability and cellular senescence-based models in predicting the one-year survival in HCC patients through a literature review. Therefore, we next investigated if these characteristics of Cu-cluster B could aid in identifying new therapeutic strategies for the treatment of patients with HCC. Therefore, the effects of anticancer drugs like a PARP inhibitor (Rucaparib), Rebemadlin, and Serdemetan that act primarily on p53 were investigated [62,63]. The patients in Cu-cluster B were less sensitive to anticancer drugs like Rucaparib, Rebemadlin, and Serdemetan, consistent with the characteristics of Cu-cluster B, i.e., a high HRD expression and TP53 mutations. Moreover, the patients in Cu-cluster B were resistant to conventional chemotherapeutic agents like Sorafenib, Lapatinib, Erlotinib, Axitinib, and Gefitinib, which could be an underlying factor associated with a poor prognosis of the patients in Cu-cluster B. 

The patients with HCC in Cu-cluster B have poor prognoses and are chemoresistant; hence, identifying biomarkers to diagnose these patients has clinical significance [64]. WGCNA and the randomForest algorithm were used to identify the CSGSs (*CDCA8*, *MCM6*, and *NCAPG2)* from Cu-cluster B. These CSGSs, could be used for diagnosis, prognosis, and to predict chemotherapeutic responses in patients with HCC. In addition, the expression pattern of *NCAPG2* in patients from the GSE62061 dataset (an independent validation cohort) could predict Erlotinib sensitivity in patients with HCC. These results were further validated by molecular docking. Erlotinib is a small-molecule *EGFR-specific* tyrosine kinase inhibitor. The binding affinity analysis showed that Erlotinib had a favorable binding affinity (docking score of −7.2 kcal/mol) for NCAPG2, comparable to that of Erlotinib and EGFR. NCAPG2, as a subunit of the condensin II complex, is responsible for regulating microtubule kinetochore attachment during mitosis and ensuring proper chromosome segregation, indicating its critical role in cell division [65]. Jieun et al. used the CRISPR-Cas9 sgRNA library to create knockout mutations in Erlotinib-resistant human tumor cells (NCI-H820) to screen for genes associated with erlotinib sensitivity. Studies have shown that the chemical inhibitors combined with Erlotinib used for treating in vitro and in vivo models of patient-derived xenografts revealed that the treatment could induce cell death by targeting the cell cycle pathway [66]. Therefore, the cell cycle pathway could be targeted to overcome Erlotinib resistance in cancers. Further, targeting the cell cycle could be an underlying mechanism by which NCAPG2 alters Erlotinib resistance in patients with HCC. However, further studies are required to understand the role of NCAPG2 in modulating Erlotinib resistance in patients with HCC. Together, these results could aid in designing personalized therapeutic strategies for patients with HCC in clinical settings.

Novel strategies that could aid in developing personalized treatment regimens for patients with HCC were illuminated by our study. Nonetheless, there were a few limitations to our study. We obtained data from publicly available databases and performed a bioinformatic analysis. Experimental and clinical validation are necessary to further support the findings of our study. However, our study was limited by its retrospective design and lack of prospective data. However, it is important to note that multiple independent cohorts were used to perform the bioinformatic analysis, which adds credibility to our data. Hence, additional studies and clinical trials are necessary to validate our results and will aid in elucidating the underlying molecular mechanisms of cuproptosis in HCC.

## 5. Conclusions

In this study, we performed unsupervised clustering on cuproptosis regulator genes to classify patients with HCC into three cuproptosis clusters (Cu-clusters A, B, and C) characterized by different senescence microenvironments, genomic instability, and prognosis. Moreover, we identified three biomarker genes (CDCA8, MCM6, and NCAPG2) in patients with the highest genomic instability and poor prognosis grouped into a cuproptosis cluster that could be used to design treatment strategies for patients with HCC. The patients in Cu-cluster B were considered to be relatively insensitive to chemotherapy. Additionally, this study suggests that NCAPG2 may be a potential novel target for the anti-HCC effects of Erlotinib. Our study lays a foundation for the role of cuproptosis in the development and progression of cancer. Furthermore, our results could aid in developing personalized strategies for the management and treatment of patients with HCC.

## Data Availability

The datasets used and/or analyzed during the current study are available from the corresponding author upon reasonable request.

## Supplementary Materials

The following supporting information can be downloaded at: https://www.mdpi.com/article/10.3390/jcm12185767/s1, Figure S1: Flow diagram of different phases of the study; Figure S2: The nomogram predicts the probability of OS in patients with HCC. (A) Nomogram was constructed based on the stage and Cu-Riskscore to estimate the 1-, 3-, and 5-year OS of patients from the TCGA-LIHC cohort. The calibration curves show the consistency between the predicted and observed 1-, 3-, and 5-year OS at different time points in the TCGA-LIHC cohort. The estimated survivals are shown on the X-axis, and the actual outcomes are shown on the Y-axis. The gray 45-degree dotted line represents the ideal calibration mode; Figure S3: (A) Differences in expression of the cuproptosis regulator genes in patients in different cuproptosis clusters. The asterisks represent the statistical *p*-values (* *p* < 0.05; ** *p* < 0.01; *** *p* < 0.001; **** *p* < 0.0001, Kruskal–Wallis test). (B) The proportions of different cuproptosis subtypes in patients with early and advanced stage HCC in the TCGA-LIHC cohort. (C) The proportions of different cuproptosis subtypes in different T stages in the TCGA-LIHC cohort. The *p*-values were calculated by the chi-square test. Based on the median expression of GLS and CDKN2A, the patients were divided into GLS (low) CDKN2A (low), GLS (low) CDKN2A (high), GLS (high) CDKN2A (low), and GLS (high) CDKN2A (high) groups. (D) Kaplan–Meier survival curves of patients in a different group from the TCGA-LIHC cohort. (E) The prognosis of patients in the GLS (high) CDKN2A (high) group was poor (HR = 2.35; log-rank *p* < 0.0001). An unsupervised clustering analysis was performed to identify different cuproptosis subtypes in patients from the ICGC-LIRI-JP (F) and GSE76427 (G) cohorts. Kaplan–Meier survival curves of patients in different cuproptosis clusters from the ICGC-LIRI-JP (H) and GSE76427 (I) cohorts. Figure S4: (A) The frequency of mutations in the top 15 genes in different cuproptosis clusters. HRD scores (B) and LOH scores (C) of different cuproptosis clusters. (D) Boxplot for the relative expression of HRD-related genes among patients in the three cuproptosis clusters, and the *p*-values are above the boxplots. Figure S5: Metascape was used to create a network of the terms enriched by hub genes in the turquoise module. Colored by cluster ID (A) and *p*-values (B). Figure S6: The expression of the CDCA8, MCM6, and NCAPG2 genes in HCC and normal tissues retrieved from the GEPIA database (red color represents tumor tissue, and black color represents normal tissue; a red asterisk represents *p* ≤ 0.01). CDCA8, MCM6, and NCAPG2 expressions were compared in patients with HCC at different tumor stages. Kaplan–Meier survival curves (OS) for the expression of CDCA8, MCM6, and NCAPG2 (*p*-values were calculated using the log-rank test). Immunohistochemistry shows the expression patterns of the CDCA8, MCM6, and NCAPG2 proteins retrieved from the HPA database. Figure S7: Differences in the IC50 values of Sorafenib, Lapatinib, Erlotinib, Axitinib, Gefitinib, Rucaparib (AG.014699), Rebemadlin (Nutlin.3a), and Serdemetan (JNJ.26854165) in patients in the high and low CDCA8, MCM6, and NCAPG2 expression groups were calculated using the “pRRophetic” R package. Figure S8: (A) ROC curve shows the relative expression of CDCA8, MCM6, and NCAPG2 in predicting Erlotinib resistance in patients from GSE62061. (B) The proportions of Erlotinib-sensitive and -resistant patients in the high and low NCAPG2 expression groups. Molecular docking results and the best pose of Erlotinib that fits the NCAPG2 protein. The docking results were visualized in both 3D (C) and 2D poses (D). Table S1: The senescent-associated secretory phenotype or the senescence messaging secretome secreted by senescent cells. Table S2: Gene set membership of 22 biological processes obtained from previous studies and the KEGG database. Table S3: The HRD and LOH scores of all patients in the TCGA-LIHC cohort were obtained from the study of Vésteinn et al. (doi:10.1016/j.immuni.2018.03.023). Table S4: The list of genes clustered in different modules. Table S5: The genes with |MM| > 0.8 and |GS| > 0.1 were selected as the hub genes in the turquoise module. Table S6: The results of the functional enrichment analysis of the hub genes in the turquoise module. Table S7: Prognostic models constructed based on cuproptosis, genomic instability, and senescence in the previous literature. Reference [67] is cited in the supplementary materials.

## References

[1] Ji Y., Chen H., Gow W., Ma L., Jin Y., Hui B., Yang Z., Wang Z. (2020). Potential biomarkers Ang II/AT1R and S1P/S1PR1 predict the prognosis of hepatocellular carcinoma. Oncol. Lett..

[2] Dhanasekaran R. (2021). Deciphering Tumor Heterogeneity in Hepatocellular Carcinoma (HCC)—Multi-Omic and Singulomic Approaches. Semin. Liver Dis..

[3] The Cancer Genome Atlas Research Network (2017). Comprehensive and Integrative Genomic Characterization of Hepatocellular Carcinoma. Cell.

[4] Zhu C., Ho Y.-J., Salomao M.A., Dapito D.H., Bartolome A., Schwabe R.F., Lee J.-S., Lowe S.W., Pajvani U.B. (2021). Notch activity characterizes a common hepatocellular carcinoma subtype with unique molecular and clinicopathologic features. J. Hepatol..

[5] Tsvetkov P., Coy S., Petrova B., Dreishpoon M., Verma A., Abdusamad M., Rossen J., Joesch-Cohen L., Humeidi R., Spangler R.D. (2022). Copper induces cell death by targeting lipoylated TCA cycle proteins. Science.

[6] Tang D., Chen X., Kroemer G. (2022). Cuproptosis: A copper-triggered modality of mitochondrial cell death. Cell Res..

[7] Meury M., Knop M., Seebeck F.P. (2017). Structural Basis for Copper-Oxygen Mediated C-H Bond Activation by the Formylglycine-Generating Enzyme. Angew. Chem. Int. Ed..

[8] Pal A. (2014). Copper toxicity induced hepatocerebral and neurodegenerative diseases: An urgent need for prognostic biomarkers. Neurotoxicology.

[9] Li Y. (2020). Copper homeostasis: Emerging target for cancer treatment. IUBMB Life.

[10] Pavithra V., Sathisha T.G., Kasturi K., Mallika D.S., Amos S.J., Ragunatha S. (2015). Serum Levels of Metal Ions in Female Patients with Breast Cancer. J. Clin. Diagn. Res..

[11] Zhang M., Shi M., Zhao Y. (2018). Association between serum copper levels and cervical cancer risk: A meta-analysis. Biosci. Rep..

[12] Yaman M., Kaya G., Simsek M. (2007). Comparison of trace element concentrations in cancerous and noncancerous human endometrial and ovary tissues. Int. J. Gynecol. Cancer.

[13] Zhang X., Yang Q. (2018). Association between serum copper levels and lung cancer risk: A meta-analysis. J. Int. Med. Res..

[14] Grubman A., White A.R. (2014). Copper as a key regulator of cell signalling pathways. Expert Rev. Mol. Med..

[15] Blockhuys S., Wittung-Stafshede P. (2017). Roles of Copper-Binding Proteins in Breast Cancer. Int. J. Mol. Sci..

[16] Cox C., Merajver S.D., Yoo S., Dick R.D., Brewer G.J., Lee J.S.-J., Teknos T.N. (2003). Inhibition of the Growth of Squamous Cell Carcinoma by Tetrathiomolybdate-Induced Copper Suppression in a Murine Model. Arch. Otolaryngol. Neck Surg..

[17] Cox C., Teknos T.N., Barrios M., Brewer G.J., Dick R.D., Merajver S.D., Bs R.D.D. (2001). The Role of Copper Suppression as an Antiangiogenic Strategy in Head and Neck Squamous Cell Carcinoma. Laryngoscope.

[18] Zhang Z., Wang H., Yan M., Wang H., Zhang C. (2017). Novel copper complexes as potential proteasome inhibitors for cancer treatment. Mol. Med. Rep..

[19] Weinstein J.N., Collisson E.A., Mills G.B., Shaw K.R.M., Ozenberger B.A., Ellrott K., Shmulevich I., Sander C., Stuart J.M., The Cancer Genome Atlas Research Network (2013). The Cancer Genome Atlas Pan-Cancer analysis project. Nat. Genet..

[20] Clough E., Barrett T. (2016). The Gene Expression Omnibus Database. Statistical Genomics.

[21] Campbell P.J., Getz G., The ICGC/TCGA Pan-Cancer Analysis of Whole Genomes Consortium (2020). Pan-cancer analysis of whole genomes. Nature.

[22] Coppé J.-P., Desprez P.-Y., Krtolica A., Campisi J. (2010). The Senescence-Associated Secretory Phenotype: The Dark Side of Tumor Suppression. Annu. Rev. Pathol. Mech. Dis..

[23] Wilkerson M.D., Hayes D.N. (2010). ConsensusClusterPlus: A class discovery tool with confidence assessments and item tracking. Bioinformatics.

[24] Langfelder P., Horvath S. (2008). WGCNA: An R package for weighted correlation network analysis. BMC Bioinform..

[25] Wang M., Wang L., Pu L., Li K., Feng T., Zheng P., Li S., Sun M., Yao Y., Jin L. (2020). LncRNAs related key pathways and genes in ischemic stroke by weighted gene co-expression network analysis (WGCNA). Genomics.

[26] Zhou Y., Zhou B., Pache L., Chang M., Khodabakhshi A.H., Tanaseichuk O., Benner C., Chanda S.K. (2019). Metascape provides a biologist-oriented resource for the analysis of systems-level datasets. Nat. Commun..

[27] Zhang B., Wu Q., Li B., Wang D., Wang L., Zhou Y.L. (2020). m6A regulator-mediated methylation modification patterns and tumor microenvironment infiltration characterization in gastric cancer. Mol. Cancer.

[28] Geeleher P., Cox N., Huang R.S. (2014). pRRophetic: An R Package for Prediction of Clinical Chemotherapeutic Response from Tumor Gene Expression Levels. PLoS ONE.

[29] Yang W., Soares J., Greninger P., Edelman E.J., Lightfoot H., Forbes S., Bindal N., Beare D., Smith J.A., Thompson I.R. (2013). Genomics of Drug Sensitivity in Cancer (GDSC): A resource for therapeutic biomarker discovery in cancer cells. Nucleic Acids Res..

[30] Engebretsen S., Bohlin J. (2019). Statistical predictions with glmnet. Clin. Epigenetics.

[31] Hanko M., Grendár M., Snopko P., Opšenák R., Šutovský J., Benčo M., Soršák J., Zeleňák K., Kolarovszki B. (2021). Random Forest–Based Prediction of Outcome and Mortality in Patients with Traumatic Brain Injury Undergoing Primary Decompressive Craniectomy. World Neurosurg..

[32] Zhang Y., Li H., Zhang W., Che Y., Bai W., Huang G. (2018). LASSO-based Cox-PH model identifies an 11-lncRNA signature for prognosis prediction in gastric cancer. Mol. Med. Rep..

[33] Gelbard R.B., Hensman H., Schobel S., Khatri V., Tracy B.M., Dente C.J., Buchman T., Kirk A., Elster E. (2019). Random forest modeling can predict infectious complications following trauma laparotomy. J. Trauma Inj. Infect. Crit. Care.

[34] Navani S. (2016). Manual evaluation of tissue microarrays in a high-throughput research project: The contribution of Indian surgical pathology to the Human Protein Atlas (HPA) project. Proteomics.

[35] Chan B.K.C. (2018). Data Analysis Using R Programming. Biostat. Hum. Genet. Epidemiol..

[36] Rich J.T., Neely J.G., Paniello R.C., Voelker C.C.J., Nussenbaum B., Wang E.W. (2010). A practical guide to understanding Kaplan-Meier curves. Otolaryngol. Neck Surg..

[37] Mandrekar J.N. (2010). Receiver Operating Characteristic Curve in Diagnostic Test Assessment. J. Thorac. Oncol..

[38] Ly A., Marsman M., Wagenmakers E.-J. (2017). Analytic posteriors for Pearson’s correlation coefficient. Stat. Neerlandica.

[39] Groth D., Hartmann S., Klie S., Selbig J. (2013). Principal Components Analysis. Comput. Toxicol. Vol. II.

[40] Zhang Z., Ma Y., Guo X., Du Y., Zhu Q., Wang X., Duan C. (2021). FDX1 can Impact the Prognosis and Mediate the Metabolism of Lung Adenocarcinoma. Front. Pharmacol..

[41] Wang Z., Dong H., Yang L., Yi P., Wang Q., Huang D. (2021). The role of FDX1 in granulosa cell of Polycystic ovary syndrome (PCOS). BMC Endocr. Disord..

[42] Shao X., Lv N., Liao J., Long J., Xue R., Ai N., Xu D., Fan X. (2019). Copy number variation is highly correlated with differential gene expression: A pan-cancer study. BMC Med. Genet..

[43] Chen Q., Wang Y., Yang L., Sun L., Wen Y., Huang Y., Gao K., Yang W., Bai F., Ling L. (2022). PM2.5 promotes NSCLC carcinogenesis through translationally and transcriptionally activating DLAT-mediated glycolysis reprograming. J. Exp. Clin. Cancer Res..

[44] Sievers P., Hielscher T., Schrimpf D., Stichel D., Reuss D.E., Berghoff A.S., Neidert M.C., Wirsching H.-G., Mawrin C., Ketter R. (2020). CDKN2A/B homozygous deletion is associated with early recurrence in meningiomas. Acta Neuropathol..

[45] Ji L., Zhao G., Zhang P., Huo W., Dong P., Watari H., Jia L., Pfeffer L.M., Yue J., Zheng J. (2018). Knockout of MTF1 Inhibits the Epithelial to Mesenchymal Transition in Ovarian Cancer Cells. J. Cancer.

[46] Goh W.Q.J., Ow G.S., Kuznetsov V.A., Chong S., Lim Y.P. (2015). DLAT subunit of the pyruvate dehydrogenase complex is upregulated in gastric can-cer-implications in cancer therapy. Am. J. Transl. Res..

[47] Hsieh Y.-H., Hsu J.-L., Su I.-J., Huang W. (2011). Genomic instability caused by hepatitis B virus: Into the hepatoma inferno. Front. Biosci..

[48] Scalise J.R., Poças R.C.G., Caneloi T.P., Lopes C.O., Kanno D.T., Marques M.G., Valdivia J.C.M., Maximo F.R., Pereira J.A., Ribeiro M.L. (2016). DNA Damage Is a Potential Marker for TP53 Mutation in Colorectal Carcinogenesis. J. Gastrointest. Cancer.

[49] Lindemann A., Takahashi H., Patel A., Osman A., Myers J. (2018). Targeting the DNA Damage Response in OSCC with *TP*53 Mutations. J. Dent. Res..

[50] Bernard E., Nannya Y., Hasserjian R.P., Devlin S.M., Tuechler H., Medina-Martinez J.S., Yoshizato T., Shiozawa Y., Saiki R., Malcovati L. (2020). Implications of TP53 allelic state for genome stability, clinical presentation and outcomes in myelodysplastic syndromes. Nat. Med..

[51] Stover E.H., Fuh K., Konstantinopoulos P.A., Matulonis U.A., Liu J.F. (2020). Clinical assays for assessment of homologous recombination DNA repair deficiency. Gynecol. Oncol..

[52] Wandt V.K., Winkelbeiner N., Bornhorst J., Witt B., Raschke S., Simon L., Ebert F., Kipp A.P., Schwerdtle T. (2021). A matter of concern—Trace element dyshomeostasis and genomic stability in neurons. Redox Biol..

[53] Finke H., Winkelbeiner N., Lossow K., Hertel B., Wandt V.K., Schwarz M., Pohl G., Kopp J.F., Ebert F., Kipp A. (2020). Effects of a Cumulative, Suboptimal Supply of Multiple Trace Elements in Mice: Trace Element Status, Genomic Stability, Inflammation, and Epigenetics. Mol. Nutr. Food Res..

[54] López-Otín C., Blasco M.A., Partridge L., Serrano M., Kroemer G. (2013). The Hallmarks of Aging. Cell.

[55] Yoshioka K., Matsuno Y. (2020). Genomic destabilization and its associated mutagenesis increase with senescence-associated phenotype expression. Cancer Sci..

[56] Lopes-Paciencia S., Saint-Germain E., Rowell M.-C., Ruiz A.F., Kalegari P., Ferbeyre G. (2019). The senescence-associated secretory phenotype and its regulation. Cytokine.

[57] Boilan E., Winant V., Dumortier E., Piret J.-P., Bonfitto F., Osiewacz H.D., Debacq-Chainiaux F., Toussaint O. (2013). Role of p38MAPK and oxidative stress in copper-induced senescence. Age.

[58] Matos L., Gouveia A.M., Almeida H. (2017). Resveratrol Attenuates Copper-Induced Senescence by Improving Cellular Proteostasis. Oxidative Med. Cell. Longev..

[59] Munk R., Panda A.C., Grammatikakis I., Gorospe M., Abdelmohsen K. (2017). Senescence-Associated MicroRNAs. Int. Rev. Cell Mol. Biol..

[60] Mayer R.L., Schwarzmeier J.D., Gerner M.C., Bileck A., Mader J.C., Meier-Menches S.M., Gerner S.M., Schmetterer K.G., Pukrop T., Reichle A. (2018). Proteomics and metabolomics identify molecular mechanisms of aging potentially predisposing for chronic lymphocytic leukemia. Mol. Cell. Proteom..

[61] Tong Y., Guo D., Lin S.-H., Liang J., Yang D., Ma C., Shao F., Li M., Yu Q., Jiang Y. (2021). SUCLA2-coupled regulation of GLS succinylation and activity counteracts oxidative stress in tumor cells. Mol. Cell.

[62] Crane E.K., Kwan S.-Y., Izaguirre D.I., Tsang Y.T.M., Mullany L.K., Zu Z., Richards J.S., Gershenson D.M., Wong K.-K. (2015). Nutlin-3a: A Potential Therapeutic Opportunity for TP53 Wild-Type Ovarian Carcinomas. PLoS ONE.

[63] Lai Z.-Y., Tsai K.-Y., Chang S.-J., Chuang Y.-J. (2021). Gain-of-Function Mutant TP53 R248Q Overexpressed in Epithelial Ovarian Carcinoma Alters AKT-Dependent Regulation of Intercellular Trafficking in Responses to EGFR/MDM2 Inhibitor. Int. J. Mol. Sci..

[64] Ikeda M., Morizane C., Ueno M., Okusaka T., Ishii H., Furuse J. (2018). Chemotherapy for hepatocellular carcinoma: Current status and future perspectives. Jpn. J. Clin. Oncol..

[65] Zhan P., Xi G.-M., Zhang B., Wu Y., Liu H.-B., Liu Y.-F., Xu W.-J., Zhu Q., Cai F., Zhou Z.-J. (2017). NCAPG2 promotes tumour proliferation by regulating G2/M phase and associates with poor prognosis in lung adenocarcinoma. J. Cell. Mol. Med..

[66] Lee J., Choi A., Cho S., Jun Y., Na D., Lee A., Jang G., Kwon J.Y., Kim J., Lee S. (2021). Genome-scale CRISPR screening identifies cell cycle and protein ubiquitination processes as druggable targets for erlotinib-resistant lung cancer. Mol. Oncol..

[67] Thorsson V., Gibbs D.L., Brown S.D., Wolf D., Bortone D.S., Yang T.H.O., Porta-Pardo E., Gao G.F., Plaisier C.L., Eddy J.A. (2018). The immune landscape of cancer. Immunity.

