# Supplementary material for: Novel Molecular Subtyping Scheme Based on In Silico Analysis of Cuproptosis Regulator Gene Patterns Optimizes Survival Prediction and Treatment of Hepatocellular Carcinoma
Source: J Clin Med. 2023 Sep 5;12(18):5767. doi: 10.3390/jcm12185767 (PMC10531788; doi:10.3390/jcm12185767)
Supplement: Supplementary file 1 [file jcm-12-05767-s001.zip › jcm-2231573-supplementary.pdf]

Figure Legends

Supplementary Figure S1:

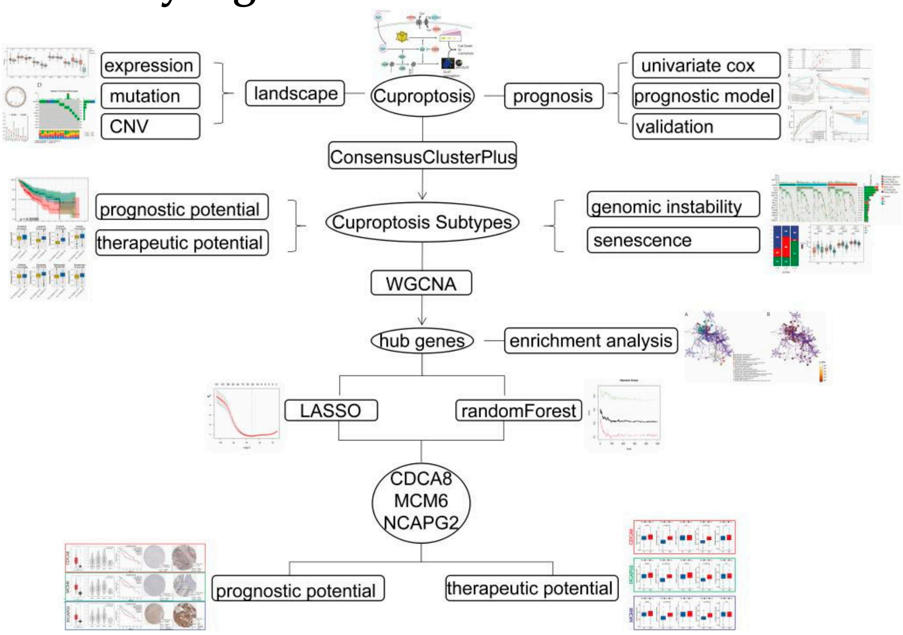

Supplementary Figure S1.

Flow diagram of different phases of the study.

Supplementary Figure S2:

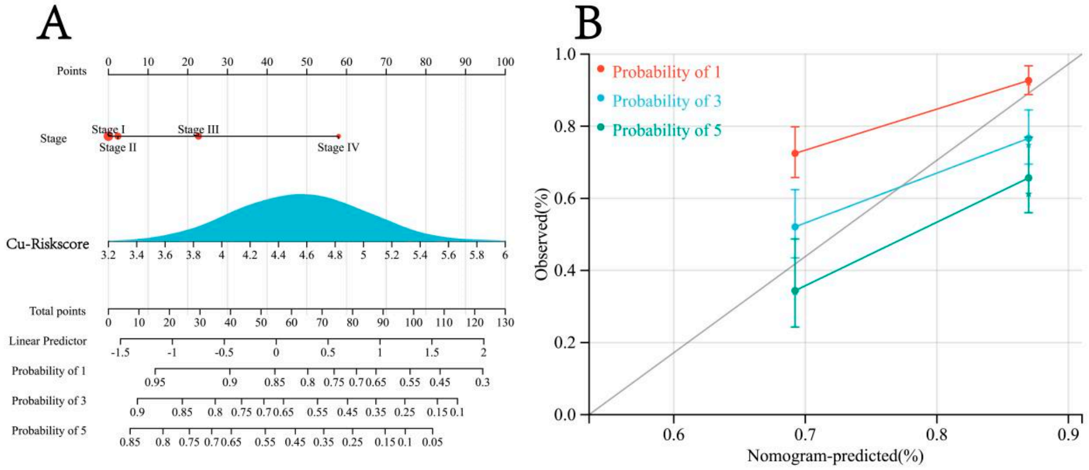

Supplementary Figure S2.

The nomogram predicts the probability of OS in patients with HCC. (A) Nomogram was constructed based on the stage and Cu−Riskscore to estimate the 1−, 3−, and 5−year OS of patients from the TCGA−LIHC cohort. The calibration curves show the consistency between the predicted and observed 1−, 3−, and 5−year OS at different time points in the TCGA−LIHC cohort. The estimated survivals are shown on the X−axis, and the actual outcomes are shown on the Y−axis. The gray 45−degree dotted line represents the ideal calibration mode.

Supplementary Figure S3:

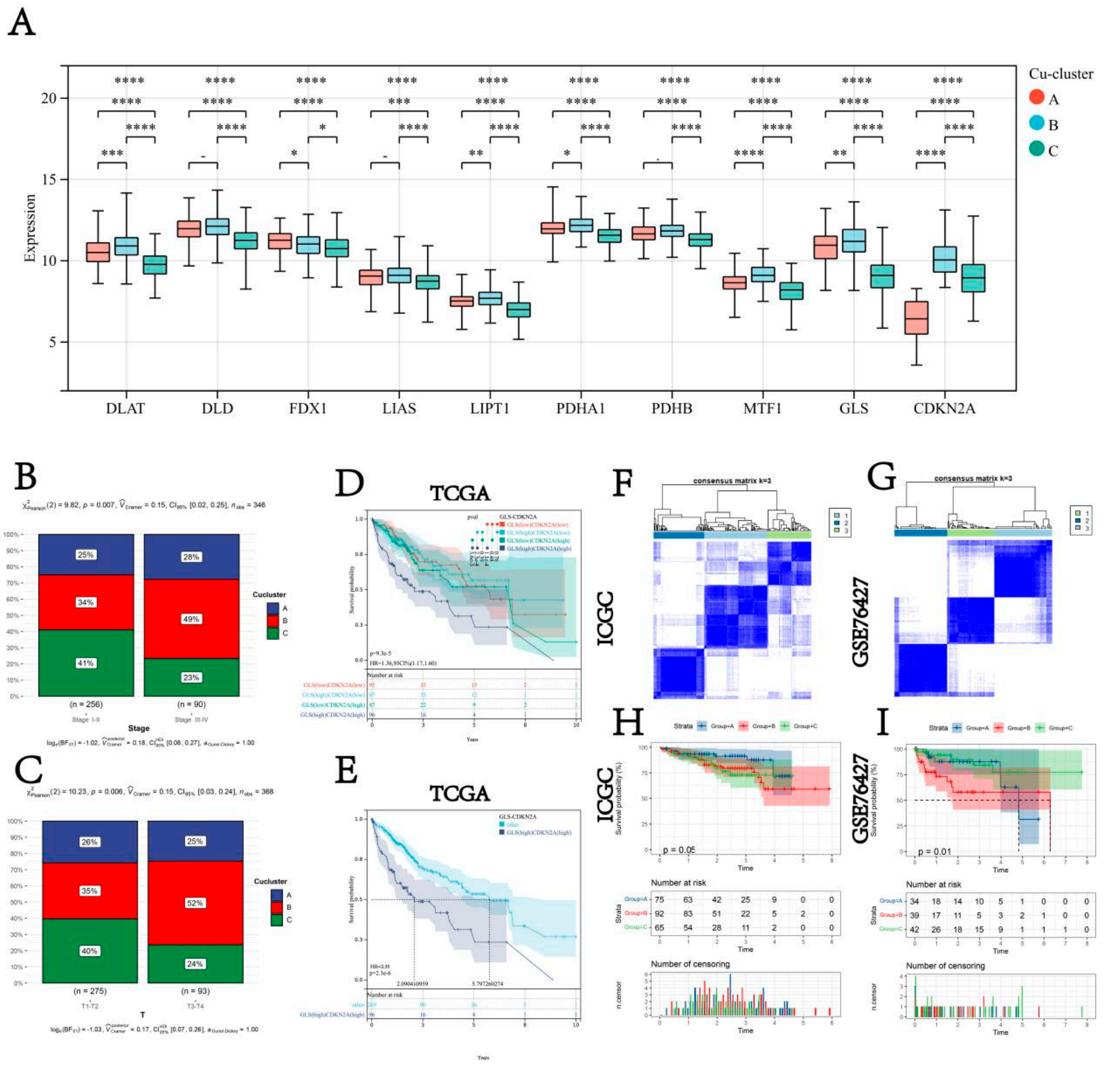

Supplementary Figure S3.

(A) Differences in expression of the cuproptosis regulator genes in patients in different cuproptosis clusters. The asterisks represent the statistical p−values (*$p < 0.05$; **$p < 0.01$; ***$p < 0.001$; ****$p < 0.0001$, Kruskal–Wallis test). (B) The proportions of different cuproptosis subtypes in patients with early and advanced stage HCC in the TCGA−LIHC cohort. (C) The proportions of different cuproptosis subtypes in different T stages in the TCGA−LIHC cohort. The p−values were calculated by the chi−square test. Based on the median expression of GLS and CDKN2A, the patients were divided into GLS (low) CDKN2A (low), GLS (low) CDKN2A (high), GLS (high) CDKN2A (low), and GLS (high) CDKN2A (high) groups. (D) Kaplan–Meier survival curves of patients in a different group from the TCGA−LIHC cohort. (E) The prognosis of patients in the GLS (high) CDKN2A (high) group was poor (HR = 2.35; log−rank p ＜ 0.0001). An unsupervised clustering analysis was performed to identify different cuproptosis subtypes in patients from the ICGC −LIRI−JP (F) and GSE76427 (G) cohorts. Kaplan–Meier survival curves of patients in different cuproptosis clusters from the ICGC−LIRI−JP (H) and GSE76427 (I) cohorts.

Supplementary Figure S4:

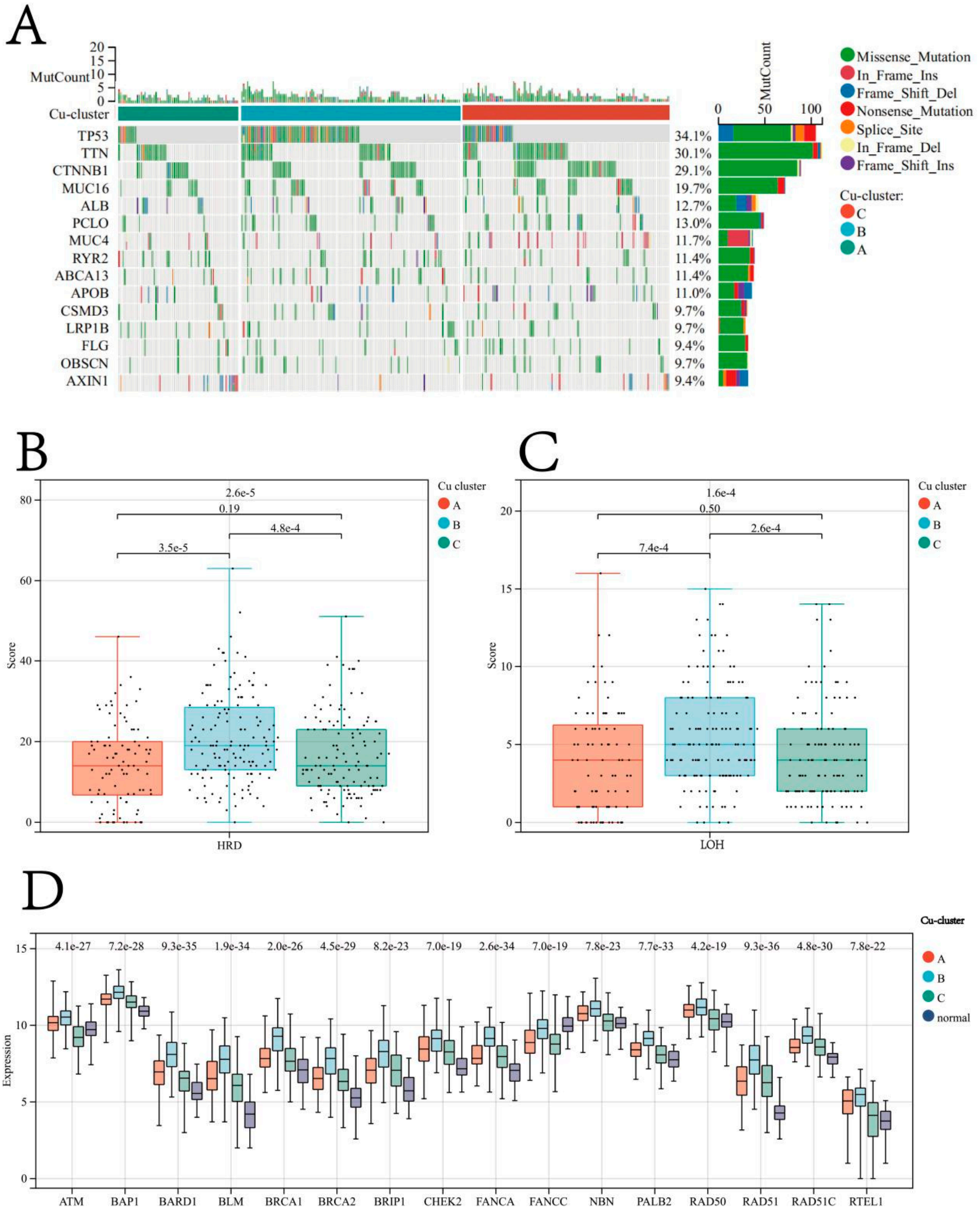

Supplementary Figure S4.

The frequency of mutations in the top 15 genes in different cuproptosis clusters. HRD scores (B) and LOH scores (C) of different cuproptosis clusters. (D) Boxplot for the relative expression of HRD−related genes among patients in the three cuproptosis clusters, and the p−values are above the boxplots.

Supplementary Figure S5:

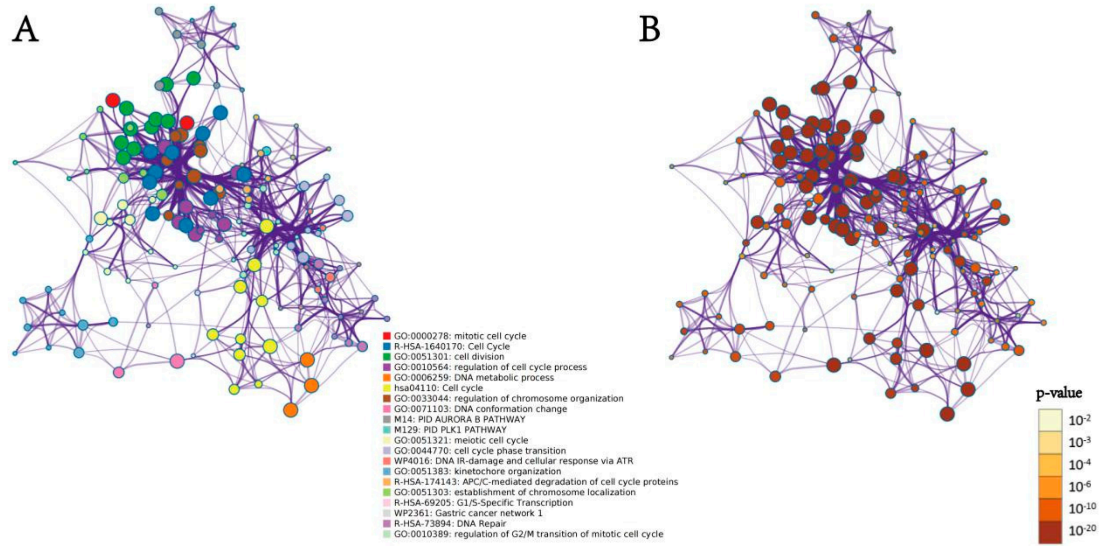

Supplementary Figure S5.

Metascape was used to create a network of the terms enriched by hub genes in the turquoise module. Colored by cluster ID (A) and $p-$values (B).

Supplementary Figure S6:

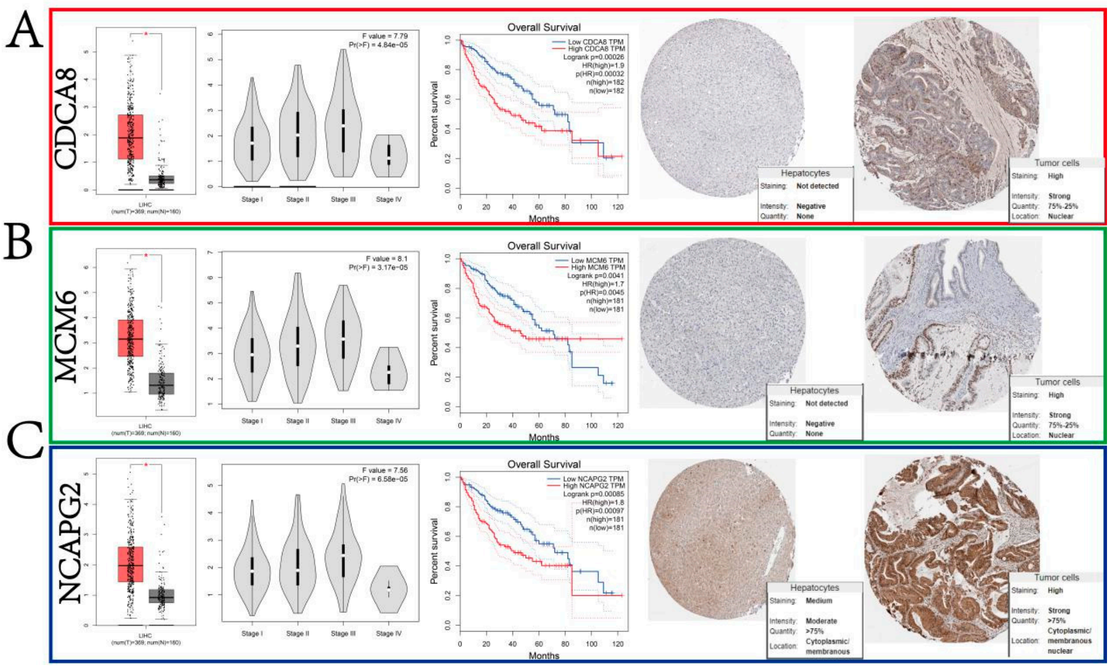

Supplementary Figure S6.

The expression of the *CDCA8, MCM6,* and *NCAPG2* genes in HCC and normal tissues retrieved from the GEPIA database (red color represents tumor tissue, and black color represents normal tissue; a red asterisk represents $p \leq 0.01$). *CDCA8, MCM6,* and *NCAPG2* expressions were compared in patients with HCC at different tumor stages. Kaplan–Meier survival curves (OS) for the expression of *CDCA8, MCM6*, and *NCAPG2* ($p-$values were calculated using the log−rank test). Immunohistochemistry shows the expression patterns of the CDCA8, MCM6, and NCAPG2 proteins retrieved from the HPA database.

Supplementary Figure S7:

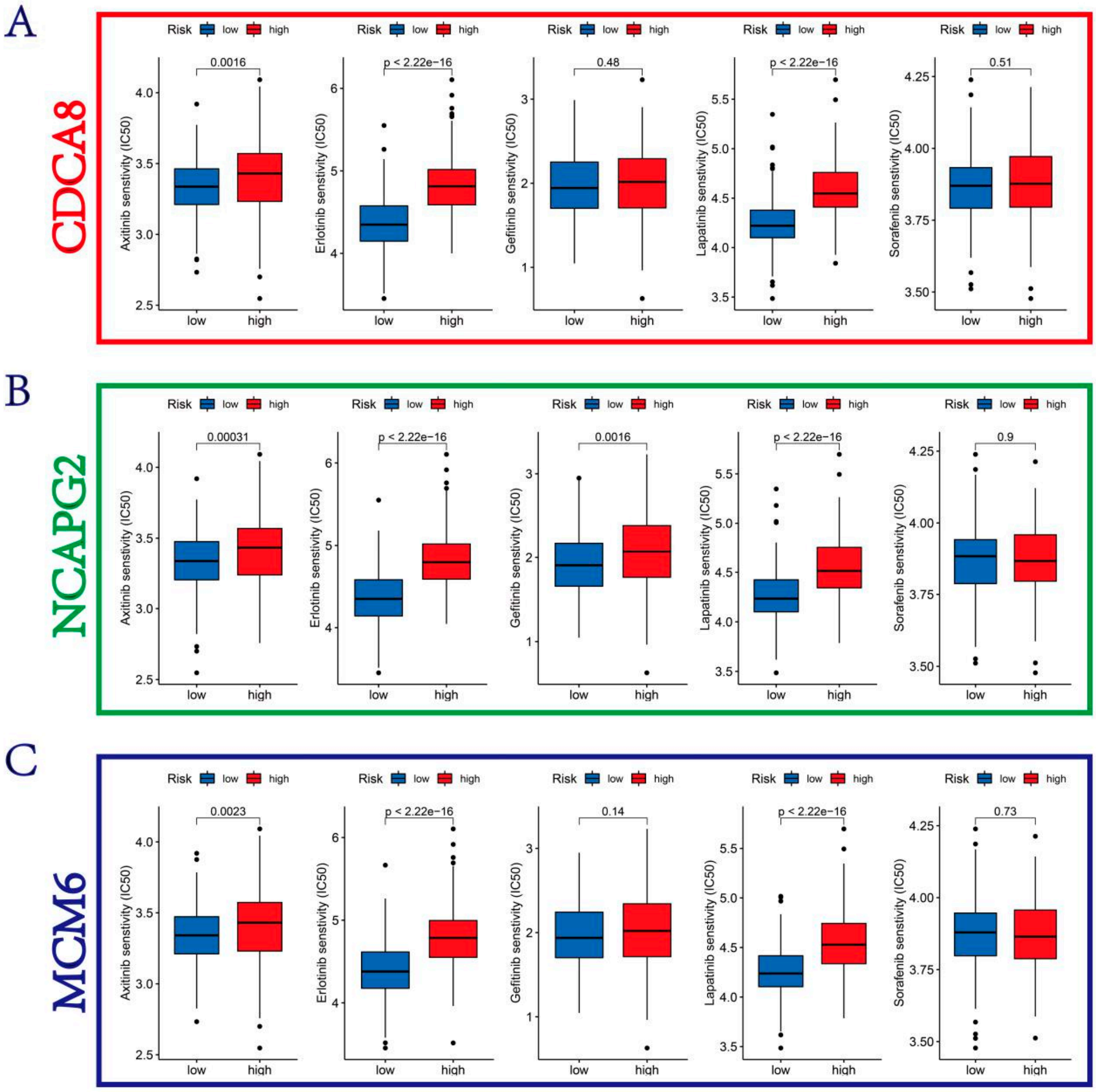

Supplementary Figure S7.

Differences in the IC_{50} values of Sorafenib, Lapatinib, Erlotinib, Axitinib, Gefitinib, Rucaparib (AG.014699), Rebemadlin (Nutlin.3a), and Serdemetan (JNJ.26854165) in patients in the high and low *CDCA8, MCM6,* and *NCAPG2* expression groups were calculated using the “pRRophetic” R package.

Supplementary Figure S8:

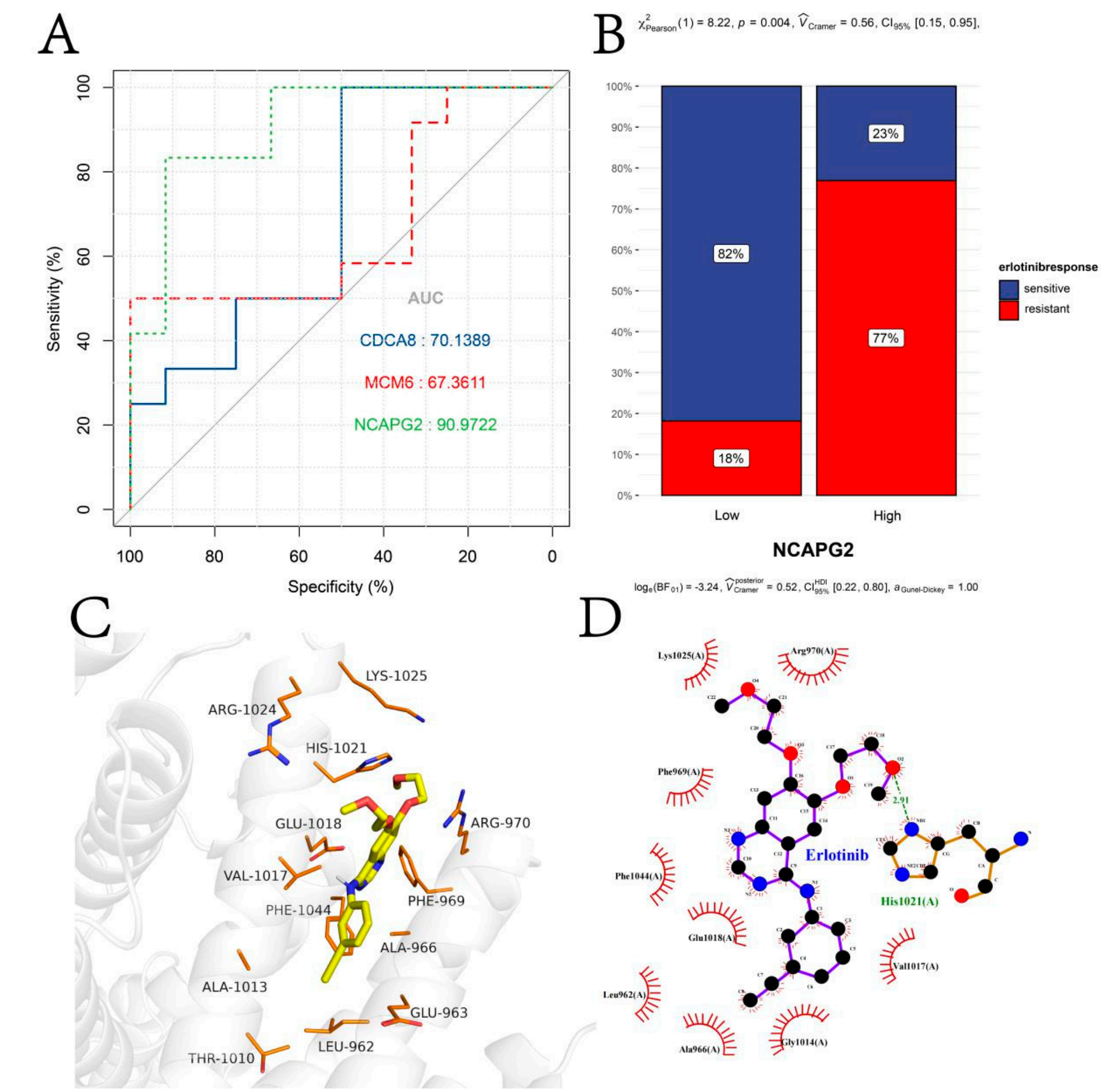

Supplementary Figure S8.

(A) ROC curve shows the relative expression of *CDCA8, MCM6,* and *NCAPG2* in predicting Erlotinib resistance in patients from GSE62061. (B) The proportions of Erlotinib−sensitive and −resistant patients in the high and low *NCAPG2* expression groups. Molecular docking results and the best pose of Erlotinib that fits the NCAPG2 protein. The docking results were visualized in both 3D (C) and 2D poses (D).

Supplementary Table S1.

The senescent−associated secretory phenotype or the senescence messaging secretome secreted by senescent cells.

Supplementary Table S2.

Gene set membership of 22 biological processes obtained from previous studies and the KEGG database.

Supplementary Table S3.

The HRD and LOH scores of all patients in the TCGA−LIHC cohort were obtained from the study of Vésteinn et al. (doi:10.1016/j.immuni.2018.03.023).

Supplementary Table S4.

The list of genes clustered in different modules.

Supplementary Table S5.

The genes with |MM| > 0.8 and |GS| > 0.1 were selected as the hub genes in the turquoise module.

Supplementary Table S6.

The results of the functional enrichment analysis of the hub genes in the turquoise module.

Supplementary Table S7.

Prognostic models constructed based on cuproptosis, genomic instability, and senescence in the previous literature.
